# Noninvasive imaging of the tumor immune microenvironment correlates with response to immunotherapy in gastric cancer

**DOI:** 10.1038/s41467-022-32816-w

**Published:** 2022-08-30

**Authors:** Weicai Huang, Yuming Jiang, Wenjun Xiong, Zepang Sun, Chuanli Chen, Qingyu Yuan, Kangneng Zhou, Zhen Han, Hao Feng, Hao Chen, Xiaokun Liang, Shitong Yu, Yanfeng Hu, Jiang Yu, Yan Chen, Liying Zhao, Hao Liu, Zhiwei Zhou, Wei Wang, Wei Wang, Yikai Xu, Guoxin Li

**Affiliations:** 1 Department of General Surgery, Nanfang Hospital, The First School of Clinical Medicine, Southern Medical University, Guangzhou, Guangdong 510515 China; 2 Guangdong Provincial Key Laboratory of Precision Medicine for Gastrointestinal Tumor, Nanfang Hospital, The First School of Clinical Medicine, Southern Medical University, Guangzhou, Guangdong 510515 China; 3 Department of Gastrointestinal Surgery, Guangdong Provincial Hospital of Chinese Medicine, the Second Affiliated Hospital of Guangzhou University of Chinese Medicine, Dade Road No. 111, Guangzhou, 510120 China; 4 Department of Medical Imaging Center, Nanfang Hospital, Southern Medical University, No. 1838, Guangzhou Avenue North, Guangzhou, 510515 China; 5 School of Computer and Communication Engineering, University of Science and Technology Beijing, Beijing, 100083 China; 6 Shenzhen Institutes of Advanced Technology, Chinese Academy of Sciences, Shenzhen, Guangdong 518055 China; 7 The Second Clinical Medical College, Guangzhou University of Chinese Medicine, Guangzhou, China; 8 Department of Gastric Surgery, Sun Yat-sen University Cancer Center, 651 Dongfeng Road East, Guangzhou, 510060 P. R. China; 9 State Key Laboratory of Oncology in South China, Collaborative Innovation Center for Cancer Medicine, Guangzhou, 510060 P. R. China

**Keywords:** Gastric cancer, Cancer microenvironment, Immunosuppression, Tumour biomarkers, Cancer imaging

## Abstract

The tumor immune microenvironment (TIME) is associated with tumor prognosis and immunotherapy response. Here we develop and validate a CT-based radiomics score (RS) using 2272 gastric cancer (GC) patients to investigate the relationship between the radiomics imaging biomarker and the neutrophil-to-lymphocyte ratio (NLR) in the TIME, including its correlation with prognosis and immunotherapy response in advanced GC. The RS achieves an AUC of 0.795–0.861 in predicting the NLR in the TIME. Notably, the radiomics imaging biomarker is indistinguishable from the IHC-derived NLR status in predicting DFS and OS in each cohort (HR range: 1.694–3.394, *P* < 0.001). We find the objective responses of a cohort of anti-PD-1 immunotherapy patients is significantly higher in the low-RS group (60.9% and 42.9%) than in the high-RS group (8.1% and 14.3%). The radiomics imaging biomarker is a noninvasive method to evaluate TIME, and may correlate with prognosis and anti PD-1 immunotherapy response in GC patients.

## Abstract (web summary)

Tumour microenvironment has been linked with immunotherapy response in gastric cancer. Here, the authors use CT-based radiomics to predict neutrophils-to-lymphocyte ratio and response to immunotherapy.

## Introduction

Gastric cancer (GC) is one of the most commonly diagnosed cancers and the third leading cause of cancer-related deaths worldwide^1^. Although surgery and chemotherapy have improved the survival rate of advanced GC patients, the overall survival (OS) rate of GC patients is <40%, and more than half of GC patients experience recurrence^2,3^. Recently, the treatment landscape of GC has been dramatically changed by immunotherapy, which has brought astounding success in clinical cancer treatment strategies^4–6^. Moreover, the Checkmate-649 trial reported a positive result for anti-PD-1 therapy in GC, increasing the confidence of patients and physicians in treatment decisions^7,8^. Unfortunately, although immunotherapy has benefited many patients with various tumors, patients with GC seem to have a variable benefit from immunotherapy^9^. Therefore, the need to identify innovative biomarkers for the prognosis and response to immunotherapy to improve GC treatment has long been overdue.

An increasing number of studies have highlighted that the tumor immune microenvironment (TIME) plays an important role in cancer progression and therapeutic response^10–12^. Therefore, effective evaluation of the TIME can help in the clinical prediction of prognosis and treatment efficacy. In recent years, studies have shown that tumor-infiltrating neutrophils can change lymphocyte behavior, resulting in tumor initiation, poor prognosis, and immunotherapy resistance^13^. Furthermore, the neutrophil-to-lymphocyte ratio (NLR) has been identified as a prognostic indicator for cancer risk stratification and therapy decision-making in various cancers^14,15^. Recently, Alessi et al. reported that the peripheral blood-derived NLR could be used for the prediction of outcomes following first-line treatment with pembrolizumab in non-small cell lung cancer^16^. However, NLR detection in the TIME is invasive, and as a result, most studies have focused on the NLR in peripheral blood, with only a few evaluating the NLR of the TIME, where the effective immune response is active. However, it may be difficult to translate a given NLR into a personalized prognosis or treatment decision due to the large variability in peripheral blood levels of neutrophils and lymphocytes between different individuals. Therefore, moving NLR detection from peripheral blood to intratumoral and peritumoral environments can aid accurate assessment of the prognosis and response to treatment of patients.

Radiomics is a promising method for translating computational medical images into mineable data. It has been proposed as a method complementary to biopsy for noninvasive evaluation of the tumor and the tumor immune microenvironment^17,18^. Previous studies have shown that medical images contain macroscopic, cellular, and molecular information about the tumor, which may help in understanding tumor behavior^19^. Importantly, with the increase in studies of predictive models, especially immune cell infiltration models, signatures from the peritumoral region are becoming increasingly appreciated, because peritumoral region features contain additional information on stromal inflammation and immune infiltration^20–22^. Immune cell infiltration in both the intratumoral and peritumoral regions is essential for activation of the immunotherapy response. Many studies have shown that the incorporation of intratumoral and peritumoral features can enhance the understanding of cancer biology and the characterization of spatial heterogeneity, thus leading to better clinical decisions^21,23,24^. Since the performance of the radiomics approach in clinical diagnosis, prognosis prediction, and treatment options in many types of cancers is better than that of many other routine methods, such as the current TNM staging system and prognostic biomarkers in plasma (CEA, CA19-9, and EBV DNA), the radiomics approach has gained increasing attention^25–27^. Moreover, the association between imaging features and the TIME has recently been widely explored, indicating the power of radiomics imaging biomarker in evaluating tumor-infiltrating cells^22,28,29^. However, the relationship between radiomics and the NLR in TIME is unclear.

In this study, we aimed to develop a noninvasive radiomics imaging biomarker of the NLR in the tumor immune microenvironment using the intratumoral and peritumoral features on computed tomography (CT) images and to further investigate its potential predictive power for prognosis and anti-PD-1 immunotherapy response.

## Results

### Clinicopathological characteristics

**Fig. 1:**
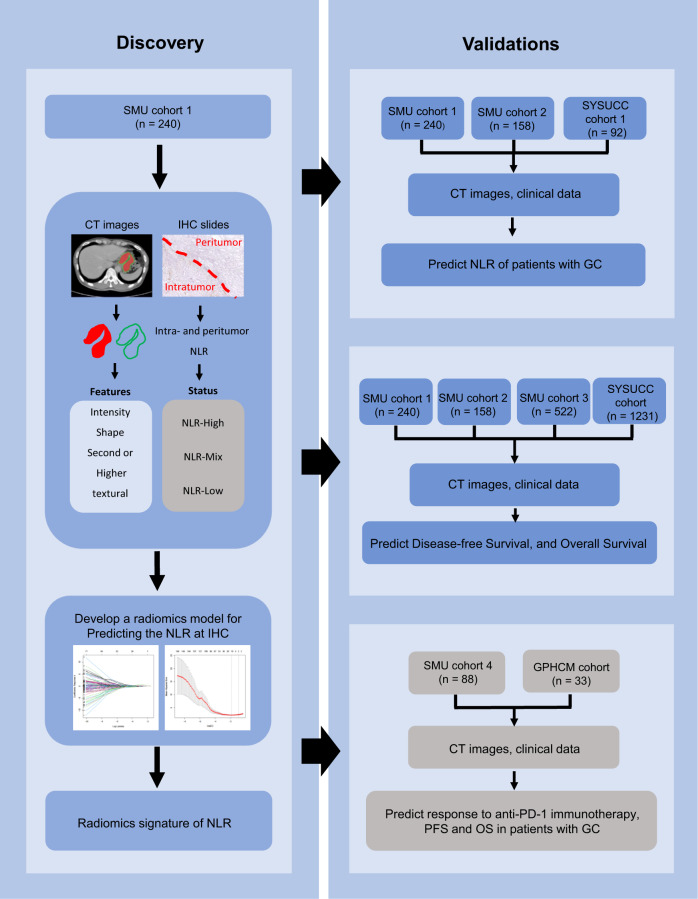
Study design for the discovery and validation of the radiomics imaging biomarker of NLR in gastric cancer. SMU cohort 1 was the training cohort for developing the radiomics imaging biomarker. SMU cohort 2 and SMU cohort 3 were the internal validation cohorts. SYSUCC cohort 1 and SYSUCC cohort were the external validation cohorts. SMU cohort 4 and the GPHCM cohort were used to evaluate the of response to anti-PD-1 immunotherapy and the clinical outcomes of immunotherapy.

This study included 2272 GC patients from 3 independent centres (Table 1 and Supplementary Table 1). Patients with NLR information or complete follow-up data (*n* = 2151) were used to predict the NLR status and survival, while those receiving immunotherapy (*n* = 121) were used to evaluate the predictive power of the radiomics imaging biomarker in predicting the response to immunotherapy and clinical outcomes of immunotherapy. The overall study design is shown in Fig. 1. The clinicopathological characteristics of the training cohort (*n* = 240), internal validation cohorts 1 (*n* = 158) and 2 (*n* = 522), and external validation cohorts 1 (*n* = 92) and 2 (*n* = 1139) are listed in Table 1. Among these patients, a total of 1470 patients (68.34%) were male, while 681 patients (31.66%) were female (median age, 57.0 (49.0–64.0) years). Most patients (*n* = 1499, 69.69%) were in stage II or III.

**Table 1:** Characteristics of patients with GC in each cohort

Variables	Training cohort (*n* = 240)		Internal validation cohort 1 (*n* = 158)		Internal validation cohort 2 (*n* = 522)		External validation cohort 1 (*n* = 92)		External validation cohort 2 (*n* = 1139)	
	*n*	%	*n*	%	*n*	%	*n*	%	*n*	%
**Gender**										
Male	151	62.9	112	70.9	361	69.2	31	33.7	354	31.1
Female	89	37.1	46	29.1	161	30.8	61	66.3	785	68.9
Age (years), median (interquartile range)	57 (49–64)		56 (46–62)		56 (48–63)		59 (45–65)		57 (50–65)	
**Differentiation**										
Well	27	11.3	10	6.3	89	17	1	1.1	19	1.7
Moderate	60	25	44	27.8	117	22.4	19	20.7	176	15.5
Poor or undifferentiation	153	63.7	104	65.9	316	60.6	72	78.2	955	82.9
**Location**										
Cardia	50	20.8	38	24.1	67	12.8	29	31.5	390	34.2
Body	47	19.6	28	17.7	95	18.2	23	25	226	19.8
Antrum	129	53.8	83	52.5	317	60.7	35	38	468	41.2
Whole	14	5.8	9	5.7	43	8.3	5	5.5	55	4.8
**Lauren type**										
Intestinal type	110	45.8	75	47.5	236	45.2	29	31.5	392	34.4
Diffuse or mixed type	130	54.2	83	52.5	286	54.8	63	68.5	747	65.6
**CEA**										
Elevated	26	10.8	20	12.7	55	10.5	16	17.4	228	20
Normal	214	89.2	138	87.3	467	89.5	76	82.6	911	80
**CA19-9**										
Elevated	31	12.9	15	9.5	88	16.9	11	12	231	20.3
Normal	209	87.1	143	90.5	434	83.1	81	88	908	79.7
**Depth of invasion**										
T1	55	22.9	27	17.1	144	27.6	14	15.2	142	12.5
T2	21	8.8	14	8.9	81	15.5	10	10.9	127	11.2
T3	29	12.1	19	12	15	2.9	21	22.8	250	21.9
T4a	110	45.8	68	43	165	31.6	38	41.3	530	46.5
T4b	25	10.4	30	19	117	22.4	9	9.8	90	7.9
**Lymph node metastasis**										
N0	110	45.8	58	36.7	245	46.9	34	37	372	32.7
N1	44	18.3	26	16.5	118	22.6	14	15.2	185	16.2
N2	22	9.2	27	17.1	62	11.9	11	12	202	17.7
N3a	35	14.6	29	18.4	70	13.4	19	20.7	246	21.6
N3b	29	12.1	18	11.4	27	5.2	14	15.2	134	11.8
**Distant metastasis**										
M(−)	233	97.1	152	96.2	475	91	84	91.3	1006	88.3
M(+)	7	2.9	6	3.8	47	9	8	8.7	133	11.7
**TNM stage**										
I	65	27.1	34	21.5	129	24.7	19	20.7	197	17.3
II	58	24.2	30	19	124	23.8	21	22.8	289	25.4
III	110	45.8	88	55.7	222	42.5	44	47.8	520	45.7
IV	7	2.9	6	3.8	47	9	8	8.7	133	11.7

*M(−)* negative metastasis, *M(+)* positive metastasis.

There were two anti-PD-1 immunotherapy cohorts (Supplementary Table 1): Nanfang Hospital of Southern Medical University (SMU) cohort (of 51 patients, 58% were male) and Guangdong Provincial Hospital of Chinese Medicine (GPHCM) cohort (of 12 patients, 36.4% were male) (median ages, 54 (46–65) years and 60 (49–66) years, respectively). All patients were in stage III or IV, except for five patients (5.7%) in the SMU cohort in stage II. Of 121 patients, 34 patients received immunotherapy as first-line treatment, 49 patients received immunotherapy as second-line treatment, and 38 patients received immunotherapy as third-line treatment. The objective response (OR: complete response and partial response) rates in the SMU cohort and GPHCM cohort were 34.1% and 21.5%, respectively.

### Association between the NLR status of the TIME and prognosis

**Fig. 2:**
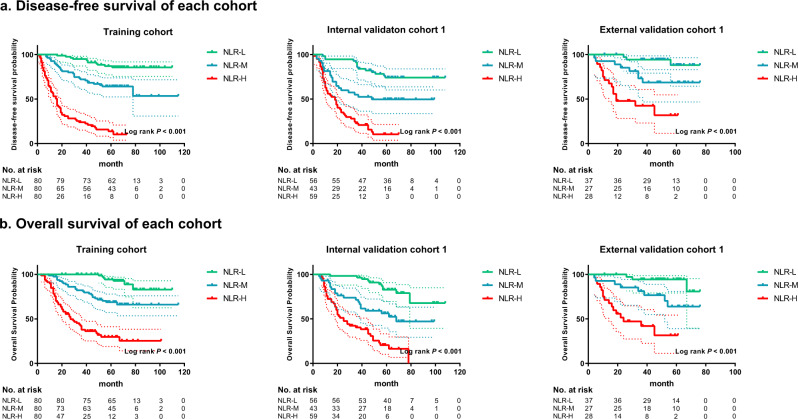
Kaplan–Meier analyses of disease-free survival (DFS) and overall survival (OS) according to different NLR statuses of the TIME in patients with gastric cancer. **a** Disease-free survival according to different NLR statuses of the TIME in the training cohort, internal validation cohort 1, and external validation cohort 1. **b** Overall survival according to different NLR statuses of the TIME in the training cohort, internal validation cohort 1, and external validation cohort 1. NLR-L: NLR-Low group, NLR-M: NLR-Mix group, NLR-H: NLR-High group, comparisons of the above progression survival curves were performed with a two-sided log-rank test. Dashed lines around the survival curves represent 95% confidence intervals. Source data are provided as a Source data file.

This study first evaluated the association between the NLR status in the TIME and prognosis. The survival curves of disease-free survival (DFS) and OS are shown in Fig. 2. The associations between the NLR status and clinicopathological variables in the training cohort, internal validation cohort 1, and external validation cohort 1 are reported in Supplementary Tables 2–4. Patients in the NLR-Low group had the best prognostic outcomes (DFS and OS) in the training cohort, internal validation cohort 1, and external validation cohort 1 (all *P* < 0.001), while those in the NLR-High group had the worst DFS and OS in each cohort (*P* < 0.001) (Fig. 2). Moreover, the survival rates were higher in the NLR-Mix group than in the NLR-High group in each cohort (*P* < 0.001). In contrast, the survival rates were lower in the NLR-Mix group than in the NLR-Low group in each cohort (*P* < 0.001) (Fig. 2). Moreover, the prognostic value of the NLR status within each subgroup of patients as defined by overall stage and other clinicopathological variables was assessed (Supplementary Figs. 1–3), and the analyses showed that NLR status was an important prognostic factor in GC. The prognosis of the subgroups (NLR-Mix 1: NLR ≥ 1 in intratumoral tissue and NLR < 1 in peritumoral tissue; NLR-Mix 2: NLR < 1 in intratumoral tissue and NLR ≥ 1 in peritumoral tissue) of the NLR-Mix group were also compared, and no significant differences were found (Supplementary Fig. 4).

### Development and validation of a radiomics imaging biomarker

**Fig. 3:**
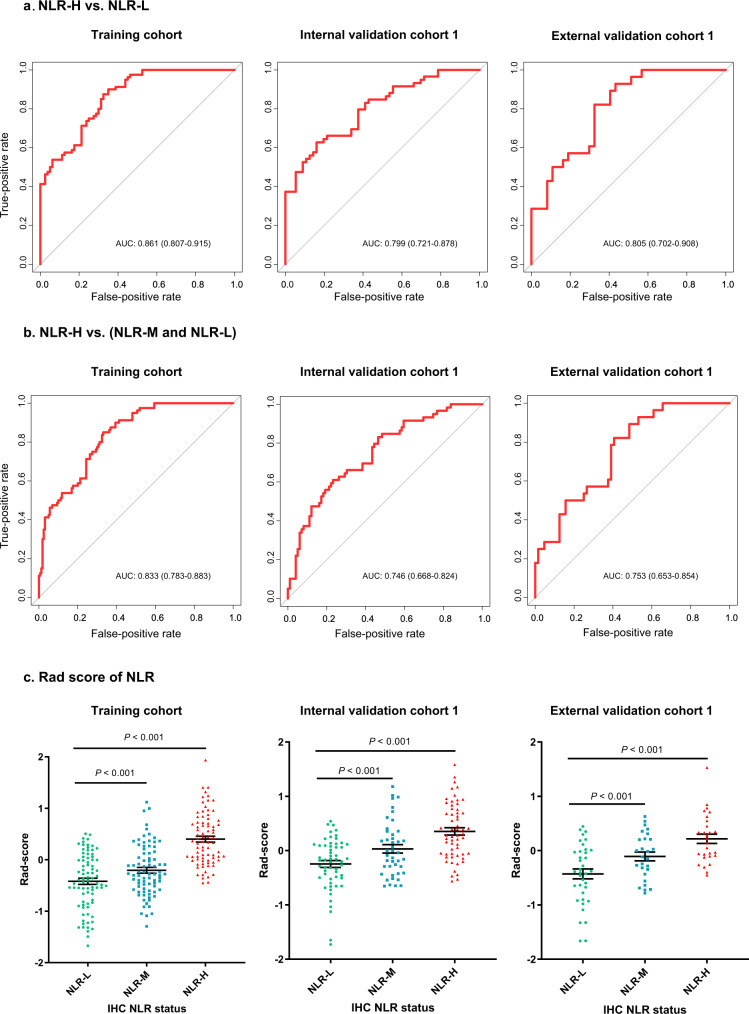
Performance of the radiomics imaging biomarker in the training and validation cohorts. **a** AUCs of the receiver operating characteristic curves of the radiomics imaging biomarker comparing the NLR-H group to the NLR-L group in each cohort. **b** AUCs of the receiver operating characteristic curves of the radiomics imaging biomarker comparing the NLR-H group to the combination of the NLR-L and NLR-M groups in each cohort. **c** Radiomics score of different NLR statuses in the training cohort (NLR-L: *n* = 80; NLR-M: *n* = 80; NLR-H: *n* = 80), internal validation cohort 1 (NLR-L: *n* = 56; NLR-M: *n* = 43, NLR-H: *n* = 59), and external validation cohort 1 (NLR-L: *n* = 37; NLR-M: *n* = 27; NLR-H: *n* = 28). The data are presented as the mean values with SEM. For statistical comparisons among different groups, a two-tailed *t* test (unpaired) was used. NLR-H: NLR-High group, NLR-M: NLR-Mix group, NLR-L: NLR-Low group. Source data are provided as a Source data file.

The maximum relevance minimum redundancy (mRMR) algorithm was used to remove the redundant features, and then, six peritumoral features and four intratumoral features were selected using least absolute shrinkage and selection operator (LASSO) regression analyses to construct a predictive radiomics imaging biomarker of the NLR in the TIME (Supplementary Fig. 5). The detailed calculation formula for the radiomics score (RS) is shown in the Supplementary Results. The associations between the radiomics imaging biomarker and clinicopathological variables are reported in Supplementary Tables 5–9. The areas under the curves (AUCs) for distinguishing the NLR-High and NLR-Low groups were 0.861 (95% CI: 0.807–0.915), 0.799 (95% CI: 0.721–0.878), and 0.805 (95% CI: 0.702–0.908) in the training cohort, internal validation cohort 1 and external validation cohort 1, respectively (Fig. 3). Moreover, the AUCs for distinguishing the NLR-High group and the combination of the NLR-Mix and NLR-Low groups in the training cohort, internal validation cohort 1 and external validation cohort 1 were 0.833 (95% CI: 0.783–0.883), 0.746 (95% CI: 0.668–0.824), and 0.753 (95% CI: 0.653–0.854), respectively (Fig. 3). We also performed ROC analysis to compare the performance of the RS and the single selected feature in predicting NLR status and found that the RS was more powerful than any individual parameter in predicting NLR status, indicating the added predictive value of the RS (Supplementary Fig. 6 and Supplementary Table 10). Importantly, the RS was significantly different among the NLR-High, NLR-Mix, and NLR-Low groups in each cohort (*P* < 0.001) (Fig. 3). The RS was significantly higher in the NLR-High groups than in the NLR-Mix and NLR-Low groups in the training cohort, internal validation cohort 1, and external validation cohort 1 (*P* < 0.001) (Fig. 3).

### Prognostic value of the radiomics imaging biomarker

**Fig. 4:**
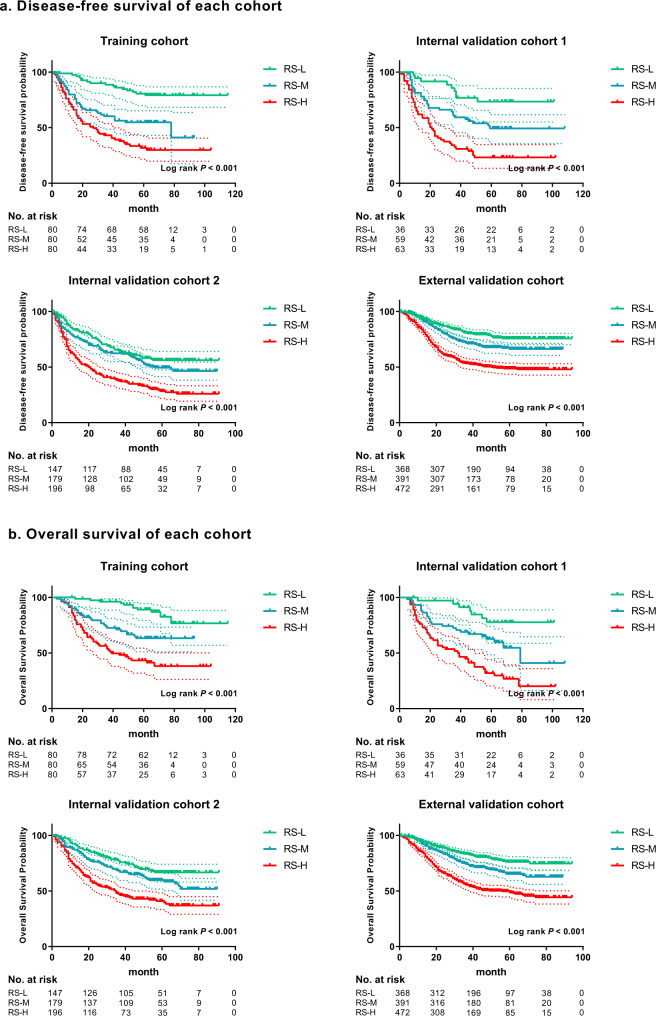
Kaplan–Meier analyses of disease-free survival (DFS) and overall survival (OS) according to different RS groups in patients with gastric cancer. **a** Disease-free survival according to different RS groups in the training cohort, internal validation cohort 1, internal validation cohort 2, and the external validation cohort. **b** Overall survival according to different RS groups in the training cohort, internal validation cohort 1, internal validation cohort 2, and the external validation cohort. RS-L: RS-Low group, RS-M: RS-Middle group, RS-H: RS-High group. Comparisons of the above progression survival curves were performed with a two-sided log-rank test. Dashed lines around the survival curves represent 95% confidence intervals. Source data are provided as a Source data file.

The radiomics imaging biomarker was significantly associated with clinical outcomes in the training cohort, internal validation cohorts 1 and 2, and the external validation cohorts 1 and 2 (Fig. 4, Tables 2 and 3). The 5-year DFS rates were higher in the RS-Low groups (79.16%, 73.47%, 56.23%, and 75.70% in the training cohort, internal validation cohorts 1 and 2, and the external validation cohort, respectively) than in the RS-High groups (31.80%, 23.24%, 28.31%, and 48.69% in the training cohort, internal validation cohorts 1 and 2 and the external validation cohort, respectively) (Fig. 4). In addition, the 5-year OS rates showed the same trend described above (Fig. 4). The 5-year OS rates in the RS-Low groups were 89.08%, 77.73%, 66.73%, and 76.13% in the training cohort, internal validation cohorts 1 and 2, and the external validation cohort, respectively. In contrast, patients in the RS-High group had the poorest 5-year OS rates (43.35% in the training cohort, 31.92% in internal validation cohort 1, 40.91% in internal validation 2, and 48.58% in the external validation cohort) (Fig. 4).

**Table 2:** Multivariate Cox regression analyses for disease-free survival and overall survival in patients with gastric cancer in the training and internal validation cohorts

Variables	Disease-free survival		Overall survival	
	HR (95% CI)	*p*	HR (95% CI)	*p*
**Training cohort**				
RS	2.118 (1.478–3.035)	<0.0001	1.894 (1.287–2.788)	0.001
**Depth of invasion**				
T1	0.068 (0.023–0.202)	<0.0001	0.069 (0.020–0.232)	<0.0001
T2	0.111 (0.035–0.358)	<0.0001	0.159 (0.049–0.515)	0.002
T3	0.233 (0.105–0.516)	<0.0001	0.206 (0.085–0.495)	<0.0001
T4a	0.273 (0.159–0.469)	<0.0001	0.258 (0.145–0.458)	<0.0001
T4b	Reference			
**Lymph node metastasis**				
N0	0.319 (0.165–0.616)	0.001	0.286 (0.137–0.597)	0.001
N1	0.415 (0.229–0.753)	0.004	0.296 (0.151–0.579)	<0.0001
N2	0.289 (0.134–0.626)	0.002	0.338 (0.147–0.778)	0.011
N3a	0.551 (0.289–1.051)	0.07	0.554 (0.273–1.121)	0.1
N3b	Reference		Reference	
Distant metastasis	3.621 (1.496–8.766)	0.004	\	\
**Internal validation cohort 1**				
RS	1.659 (1.045–2.634)	0.032	1.744 (1.103–2.757)	0.017
**Depth of invasion**				
T1	0.134 (0.040–0.444)	0.001	0.103 (0.027–0.388)	0.001
T2	0.307 (0.095–0.989)	0.048	0.277 (0.088–0.868)	0.028
T3	0.257 (0.111–0.591)	0.001	0.257 (0.111–0.594)	0.001
T4a	0.403 (0.235–0.691)	0.001	0.403 (0.228–0.713)	0.002
T4b	Reference		Reference	
**Lymph node metastasis**				
N0	0.156 (0.065–0.379)	<0.0001	0.255 (0.103–0.634)	0.003
N1	0.198 (0.087–0.451)	<0.0001	0.253 (0.102–0.628)	0.003
N2	0.321 (0.150–0.689)	0.004	0.396 (0.174–0.902)	0.027
N3a	0.407 (0.191–0.869)	0.02	0.586 (0.265–1.296)	0.187
N3b	Reference		Reference	
**Internal validation cohort 2**				
RS	1.478 (1.209–1.806)	0.0001	1.544 (1.243–1.919)	<0.0001
CEA (elevated versus normal)	1.429 (1.004–2.036)	0.048	1.720 (1.161–2.546)	0.007
CA19-9 (elevated versus normal)	1.782 (1.300–2.442)	<0.0001	1.598 (1.118–2.285)	0.01
**Depth of invasion**				
T1	0.148 (0.098–0.224)	<0.0001	0.140 (0.085–0.230)	<0.0001
T2	0.278 (0.167–0.465)	<0.0001	0.373 (0.216–0.644)	<0.0001
T3	0.427 (0.200–0.908)	0.027	0.598 (0.265–1.349)	0.215
T4a	0.405 (0.295–0.555)	<0.0001	0.477 (0.339–0.672)	<0.0001
T4b	Reference		Reference	
Distant metastasis	2.471 (1.725–3.542)	<0.0001	2.080 (1.415–3.058)	<0.0001

*P* values reported are two-tailed from Cox proportional hazard regression analyses.

*RS* radiomics score, *HR* hazard ratio.

**Table 3:** Multivariate Cox regression analyses for disease-free survival and overall survival in patients with gastric cancer in the external validation cohort

Variables	Disease-free survival		Overall survival	
	HR (95% CI)	*p*	HR (95% CI)	*p*
**External validation cohort**				
RS	1.180 (1.013–1.373)	0.033	1.167 (1.003–1.358)	0.046
Age (≥60 vs. <60 years)	1.269 (1.028–1.565)	0.026	1.263 (1.023–1.558)	0.03
**Location**				
Cardia	0.841 (0.567–1.246)	0.388	0.731 (0.496–1.976)	0.112
Body	0.467 (0.308–0.708)	<0.0001	0.443 (0.294–0.668)	<0.0001
Antrum	0.572 (0.386–0.845)	0.005	0.503 (0.342–0.740)	<0.001
Whole	Reference		Reference	
CA19-9 (elevated versus normal)	1.553 (1.242–1.943)	<0.0001	1.563 (1.248–1.957)	<0.0001
Lauren type (diffuse or mixed vs. Intestinal)	1.697 (1.238–2.326)	0.001	1.554 (1.242–1.943)	0.0001
**Depth of invasion**				
T1	0.306 (0.149–0.629)	0.001	0.265 (0.121–0.581)	0.001
T2	0.318 (0.178–0.566)	<0.0001	0.341 (0.191–0.608)	<0.0001
T3	0.442 (0.306–0.638)	<0.0001	0.445 (0.308–0.645)	<0.0001
T4a	0.779 (0.581–1.045)	0.096	0.791 (0.589–1.062)	0.119
T4b	Reference		Reference	
**Lymph node metastasis**				
N0	0.216 (0.146–0.321)	<0.0001	0.218 (0.147–0.323)	<0.0001
N1	0.428 (0.300–0.612)	<0.0001	0.413 (0.287–0.595)	<0.0001
N2	0.561 (0.408–0.770)	<0.0001	0.580 (0.421–0.799)	0.001
N3a	0.670 (0.505–0.888)	0.005	0.699 (0.526–0.928)	0.013
N3b	Reference		Reference	
Distant metastasis	2.473 (1.920–3.186)	<0.0001	2.503 (1.941–3.228)	<0.0001

*P* values reported are two-tailed from Cox proportional hazard regression analyses.

*RS* radiomics score, *HR* hazard ratio.

Univariate Cox regression analysis revealed that the radiomics imaging biomarker was a prognostic factor for DFS and OS in each cohort (Supplementary Tables 11–14). Moreover, multivariate Cox regression analysis adjusted for clinicopathological variables, including TNM stage and histologic subtype, revealed that the radiomics imaging biomarker remained an independent prognostic factor for DFS and OS in each cohort (Tables 2 and 3). We assessed the prognostic value of the radiomics imaging biomarker in each subgroup of patients as defined by clinicopathological variables. Our analyses showed significant differences in DFS and OS between patients in the three RS groups in all subgroups defined by overall stage, T stage, N stage, size, and histological subtype (Supplementary Figs. 7–9). Taken together, these data suggest that the radiomics imaging biomarker is an effective independent prognostic factor in gastric cancer.

We also investigated the relationship between clinicopathologic factors and clinical outcomes. Depth of invasion (T) and lymph node metastasis (N) were found to be significantly associated with DFS and OS (*P* < 0.001) in the training cohort. Moreover, distant metastasis was significantly associated with DFS (*P* = 0.004) in the training cohort. The variables were then integrated with the RS to develop nomograms for DFS and OS based on their contribution to different clinical outcomes (Supplementary Figs. 10 and 11). To evaluate the enhanced power of the nomograms, we then calculated the C-indices for comparing the performance. Importantly, in the training cohort, the C-indices of the nomogram for predicting DFS (0.784 (95% CI: 0.745–0.823) and OS (0.791 (95% CI: 0.749–0.834)) were higher than those of RS and TNM stage, indicating that the nomogram integrating RS and TNM stage can enhance the predictive power in predicting prognosis compared with RS or TNM stage alone (Supplementary Table 15). Similar results were found in the validation cohorts (Supplementary Table 15).

### Predictive value of the radiomics imaging biomarker for anti-PD-1 immunotherapy response

**Fig. 5:**
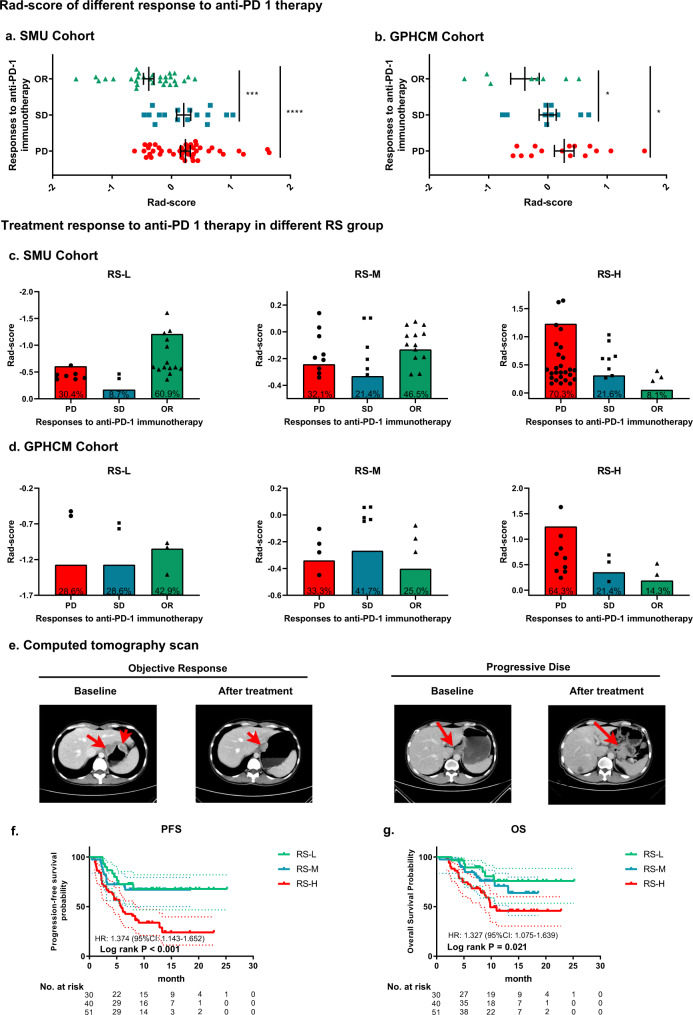
Performance of the radiomics imaging biomarker in evaluating the response to anti-PD-1 immunotherapy and clinical outcomes of immunotherapy. **a** Rad-score of different responses to anti-PD-1 immunotherapy in the SMU cohort (OR: *n* = 30; SD: *n* = 16; PD: *n* = 42). **b** Rad-score of different responses to anti-PD-1 immunotherapy in the GPHCM cohort (OR: *n* = 8; SD: *n* = 10; PD: *n* = 15); The data are presented as the mean values with SEM. For statistical comparisons among different groups, a two-tailed *t* test (unpaired) was used. **c** Rad-scores of patients and proportions of anti-PD-1 immunotherapy responses in different RS groups of the SMU cohort. **d** Rad-scores of patients and proportions of anti-PD-1 immunotherapy response in different RS groups of the GPHCM cohort. **e** CT images and changes in tumor volume of different responses to anti-PD-1 immunotherapy. **f** Progression-free survival of different RS groups in GC patients treated with anti-PD-1 immunotherapy. **g** Overall survival of different RS groups in GC patients treated with anti-PD-1 immunotherapy. Comparisons of the above progression survival curves were performed with a two-sided log-rank test. Dashed lines around the survival curves represent 95% confidence intervals. OR: objective response, SD: stable disease, PD: progressive disease, RS-L: RS-Low group, RS-M: RS-Middle group, RS-H: RS-High group, HR: hazard ratio, *P < 0.05, ***P < 0.001, ****P < 0.0001. Source data are provided as a Source data file.

This study assessed the associations between the radiomics imaging biomarker and the response to anti-PD-1 immunotherapy and the clinical outcomes of immunotherapy in the two cohorts. Interestingly, the RS was significantly lower (mean: −0.383, 95% CI (−0.565, −0.201)) in the OR group than in the progressive disease (PD) group (0.234, 95% CI (0.067, 0.402)) and the stable disease (SD) group (0.205, 95% CI (−0.048, 0.458)) in the SMU cohort (*P* < 0.001) (Fig. 5). Similar results were found in the GPHCM cohort. The mean RSs in the OR, PD and SD groups were −0.388 (95% CI: −0.958, 0.182), 0.275 (95% CI: −0.079, 0.629) and −0.003 (95% CI: −0.330, 0.324), respectively. When classifying the patients into different RS groups, we found that the OR rates (60.9% and 42.9% in the SMU and GPHCM cohorts, respectively) were significantly higher in the RS-Low group than in the RS-High group (8.1% and 14.3% in the SMU and GPHCM cohorts, respectively) (Fig. 5). Moreover, 69.6% and 71.5% of patients in the RS-Low group had disease control (stable disease, partial response, or complete response) in the SMU and GPHCM cohorts, respectively. In the RS-High group, only 29.7% and 35.7% of patients had disease control in the SMU and GPHCM cohorts, respectively (Fig. 5). In contrast, the RS-High group had the highest PD rates (70.3% and 64.3%) (Fig. 5).

Because of the limited number of patients in the GPHCM cohort, patients from the SMU cohort and GPHCM cohort were combined to evaluate progression-free survival (PFS) and OS. Notably, the median PFS in the RS-Low group was 7.95 months (95% CI: 8.02–13.04), which was longer than that in the RS-High group (median: 5.63 months, 95% CI: 5.30–8.08). Similarly, the median OS in the RS-Low group was longer than that in the RS-High group (median: 11.30 months (95% CI: 9.62–14.06) vs. 8.80 months (95% CI: 7.82–10.66)). Moreover, Kaplan–Meier analysis showed that the radiomics imaging biomarker was significantly associated with PFS (HR: 1.374 (95% CI: 1.143–1.652), *P* < 0.001) and OS (HR: 1.327 (95% CI: 1.075–1.639), *P* = 0.021) (Fig. 5). Patients in the RS-Low group had longer PFS and OS times than patients in the RS-High group (Fig. 5), indicating that the radiomics imaging biomarker is associated with the response to anti-PD-1 immunotherapy. Similar results were found after performing subgroup analysis stratified by disease stage (Supplementary Fig. 12).

Because there were different treatment lines of immunotherapy in the SMU cohort, subgroup analyses of treatment lines were performed to assess the performance of the RS in evaluating the response to anti-PD-1 immunotherapy (Supplementary Fig. 13). To our surprise, patients with a low RS still had the highest OR rates (87.5% in the first-line treatment group; 46.7% in the second or third-line treatment groups), regardless of the treatment line (Supplementary Fig. 13), further indicating that the radiomics imaging biomarker of the NLR is associated with the response to immunotherapy in GC.

## Discussion

The tumor immune microenvironment is increasingly being recognized as a major determinant of tumor biology and a regulator of antitumor drug sensitivity^30^. Tumor-infiltrated cells participate in each step of carcinogenesis from tumor initiation, and tumor growth to metastasis and treatment response. For a long time, lymphocytes were regarded as the key tumor-associated cell type in tumor progression. However, in recent years, other immune cells, such as neutrophils, which can alter the behavior of lymphocytes and therefore regulate tumor progression, have been increasingly appreciated^13^. In addition, the crucial role of the TIME in identifying patients who could benefit from immunotherapy has been recognized due to the increased exploration of immunotherapy for cancer treatment^29,31–33^. However, currently, the clinical detection of tumor-infiltrated immune cells mainly relies on immunohistochemistry (IHC) staining or flow cytometry through invasive biopsies, which have a risk of morbidities. Therefore, an effective noninvasive method for examining the TIME can help in assessing prognosis and making treatment decisions. Herein, based on a large number of pathology slides of cancer tissue, a radiomics imaging biomarker was developed using intratumoral and peritumoral features from CT images to noninvasively predict the NLR in the TIME of GC patients. Notably, the analysis conducted before biomarker development showed that the IHC-derived NLR status was strongly associated with DFS and OS, indicating the crucial role of the NLR in the TIME in prognosis. Furthermore, the results showed that the radiomics imaging biomarker could predict the NLR status in the TIME with good performance. Further analysis showed that the predictive biomarker was indistinguishable from the IHC-derived NLR status in predicting DFS and OS, indicating that the radiomics imaging biomarker could potentially be a surrogate for IHC-determined NLR detection. The results also showed that the radiomics imaging biomarker was related to the response to anti-PD-1 immunotherapy and could predict the PFS and OS of patients who received immunotherapy, indicating its potential benefit in predicting prognosis and facilitating treatment decision making.

Recently, studies on radiomics have enhanced the understanding of the role of radiomics in distinguishing tumor homogeneity and heterogeneity. A recent study reported that the combination of intratumoral and peritumoral features can effectively predict complete pathological response to neoadjuvant chemotherapy in breast cancer regardless of the receptor status^21^, indicating the clinical value of the peritumoral features. Moreover, the above study reported that a combination of peritumoral and intratumoral features is related to tumor-infiltrated lymphocytes, consistent with the findings of this study^21^. Our previous study found that radiomics features from the tumor and its periphery can be used to evaluate the immune cells in the tumor microenvironment^22^. In fact, the peritumoral area comprises the tumor-stroma interface and can be used to evaluate the immune response. Herein, this study used a cohort of IHC samples to identify the immune cells infiltrated in both the tumoral and peritumoral regions to evaluate patient prognosis and the effective immune response. Importantly, this study not only assessed the NLR in the tumor, but also assessed the NLR in the peritumoral tissues. Correspondingly, features were extracted from both tumor and its periphery. The developed radiomics imaging biomarker showed good performance in predicting the NLR in the TIME, indicating the potential predictive value of peritumoral features. Interestingly, in this study, the textures that reflect the dissimilarity and coarseness of tumors, such as NGTDM, GLCM, and GLRLM features, were strongly associated with the NLR status of the TIME. Therefore, these features may be used to differentiate the density of the neutrophils and lymphocytes in the TIME.

Many studies have reported that neutrophils and other cells, including macrophages, can stimulate the tumor microenvironment by secreting many cytokines^34–38^. Moreover, the NLR has been recognized as an important predictor of clinical outcomes and immunotherapy treatment response^16,39–41^. However, these studies mainly focused on the NLR in peripheral blood, with only a few evaluating the NLR in the TIME. Notably, an effective tumor immune response mainly occurs in the TIME. It was interesting that in this study, although an elevated NLR status in the TIME was caused by both an increase in neutrophils and a decrease in lymphocytes, the decrease in lymphocytes had the greatest impact on NLR-High status. Therefore, an elevated NLR status in the TIME may represent a “colder” TIME that suppresses antitumor function^42^. In this study, we used a radiomics-based biomarker to noninvasively predict and validate the NLR in the TIME in multiple institutions. The radiomics-based biomarker was then verified to be similar to the IHC-derived NLR status in predicting DFS and OS, suggesting that this imaging biomarker might hold potential use as a useful surrogate for biopsy in evaluating the TIME.

Moreover, the radiomics imaging biomarker was associated with the response to anti-PD-1 immunotherapy and the clinical outcomes of immunotherapy in both immunotherapy cohorts. Patients with a low RS had the highest OR rate regardless of the treatment line, indicating that the NLR status of the TIME is associated with the response to immunotherapy. In this study, the PFS and OS times were longer in the RS-Low group than in the RS-High group after treatment with immunotherapy, indicating that the radiomics imaging biomarker could be used as an additional method for predicting the benefit of immunotherapy to guide clinical practice.

In recent years, immunotherapy has benefited patients with many cancer types. Moreover, immunotherapy has been approved as an adjuvant treatment for non-small cell lung cancer, bringing a promising future for immunotherapy use to improve cancer treatment^43^. However, the current implementation of immunotherapy in GC is limited. Although immunotherapy has been recognized as a first-line treatment for advanced GC in the NCCN Clinical Practice Guidelines in Oncology, anti-PD-1 drugs are still suggested as a combination regimen with chemotherapy^44^. Anti-PD-1 or anti-PD-L1 drugs are currently recommended for cancer patients based on PD-L1 detection in tumor or immune cells via IHC, microsatellite instability (MSI) detection via polymerase chain reaction, or mismatch repair deficiency detection via IHC^45^. For GC, immunotherapy is also recommended if the patient is positive for Epstein–Barr virus (EBV)^46^. However, the detection of these markers requires invasive procedures. Moreover, some studies have reported that PD-L1 expression alone is not sufficient to predict the treatment response to immunotherapy^47,48^. Although response rates are more than 50% in MSI-high tumors, these tumors constitute only ~4% of gastroesophageal cancers; therefore, relying on the MSI status to guide treatments may limit the implementation of immunotherapy^46^. In addition, the OR rate of EBV positive GC varies from 0 to 100%, indicating that EBV is not a stable biomarker for the evaluation of immunotherapy^49,50^. Indeed, most of the patients receiving immunotherapy in this study met at least one of the above implementation criteria to use anti-PD-1 immunotherapy, but the actual OR rate was still low, and most of the patients were ultimately found to have progressive disease, indicating that the above PD-L1/MSI/EBV biomarkers are still limited in predicting the efficacy of immunotherapy. The limitation of the above markers in evaluating the efficacy of immunotherapy suggests that the response to immunotherapy does not rely on the detection of a single tumor feature or a single tumor-infiltrated immune cell type. Effective evaluation of the TIME may help in improving current therapeutic decisions. Therefore, we developed a biomarker that could not only noninvasively evaluate the TIME, but also potentially predict the potential response to immunotherapy, providing a tool which could be included in clinical decision making.

The present study found that the radiomics imaging biomarker of the NLR in the TIME of GC could be used to evaluate the response to immunotherapy regardless of the treatment line. However, due to the limited number of immunotherapy cohorts, in the future, studies on the radiomics imaging biomarker of the NLR in the TIME should further investigate the association between radiomics imaging biomarkers and the response to immunotherapy.

This study has some limitations. First, this was a retrospective study. As a result, this study included a large number of patients from three independent centres to validate the findings and ensure reproducibility. The second limitation is the difference in the quality of the CT images obtained from different scanners and institutions. Therefore, this study conducted data standardization to minimize the effect of the above issue. Third, the number of patients in the immunotherapy cohorts was limited. Anti-PD-1 drugs are widely used as second- or third-line treatments for GC and have been applied as regular drugs for just a few years, limiting the number of study patients. Therefore, since anti-PD-1 drugs are recognized as a first-line treatment for advanced GC in the NCCN Clinical Practice Guidelines in Oncology, prospective and randomized clinical trials are needed to further validate the findings of the current study.

In conclusion, this study suggests that the radiomics imaging biomarker can effectively and noninvasively predict the NLR in the TIME of GC. Furthermore, the radiomics imaging biomarker of the NLR in the TIME identified in this study is correlated with the clinical outcomes and the response to immunotherapy.

## Methods

This study was approved by the Institutional Review Board of Nanfang Hospital of Southern Medical University, Sun Yat-sen University Cancer Center, and Guangdong Provincial Hospital of Chinese Medicine. Informed consent was waived since this was a retrospective study.

### Patients

The overall study design is shown in Fig. 1. To predict NLR and survival, this study retrospectively enrolled a training cohort (*n* = 240), internal validation cohort 1 (*n* = 158), and internal validation cohort 2 (*n* = 522) from Nanfang Hospital of Southern Medical University (SMU, Guangzhou, China) (2005–2015). The external validation cohort (*n* = 1231, including external validation cohort 1 (*n* = 92) used for predicting NLR status) was retrospectively enrolled from Sun Yat-sen University Cancer Center (SYUCC, 2008–2012). To evaluate the response to anti-PD-1 immunotherapy and the clinical outcomes of immunotherapy, this study retrospectively selected patients with pathologically confirmed GC who received anti-PD-1 immunotherapy in Nanfang Hospital of Southern Medical University (*n* = 88) and Guangdong Provincial Hospital of Chinese Medicine (GPHCM, Guangzhou, China) (*n* = 33) from January 2019 to June 2021. The major inclusion and exclusion criteria are listed in the Supplementary Methods.

PFS was defined as the time from anti-PD-1/PD-L1 chemotherapy initiation to tumor progression or death from any cause. DFS was defined as the time from surgery to either disease progression or death from any cause. OS was defined as the time to death from any cause.

### Immunohistochemistry staining and definition of NLR status

Formalin-fixed paraffin-embedded samples were processed for IHC staining, as described in previous studies^51,52^. The tumor and adjacent samples were incubated with antibodies against human CD8 and CD66b to mark lymphocytes and neutrophils^13^. The NLR was defined as the neutrophil count/the lymphocyte count. The NLR status of the TIME was divided into three groups: NLR High (NLR-H: NLR ≥ 1 both in intratumoral and peritumoral tissues); NLR Mix (NLR-M: NLR ≥ 1 in intratumoral tissue and NLR < 1 in peritumoral tissue, or NLR < 1 in intratumoral tissue and NLR ≥ 1 in peritumoral tissue); NLR Low (NLR-L: NLR < 1 in both intratumoral and peritumoral tissues). Detailed information is shown in the Supplementary Methods.

### Image acquisition, processing, and feature extraction

Portal venous phase CT images were obtained from the picture archiving and communication system (PACS, Carestream Canada). The acquisition parameters are shown in the Supplementary Methods.

Two radiologists, CC and QY with 12 and 11 years of clinical experience in abdominal CT interpretation, respectively, manually segmented the CT images using ITK-SNAP software (version 3.8, www.itksnap.org). In addition to the tumor region, this study also developed a peripheral ring surrounding the primary tumor region, which automatically dilated the tumor boundaries by 2 mm on the outside and shrunk the tumor boundaries by 1 mm on the inside (a ring with a thickness of 3 mm), to extract the information of the invasive margin. Air cavities, adjacent organs, and large vessels were excluded. All CT images were processed following the Image Biomarker Standardization Initiative (IBSI) guidelines^53^.

This study extracted 584 quantitative features (292 in the peritumoral area and 292 in the intratumoral area) of each region of interest (ROI). The feature pool had 14 first-order intensity features, eight shape features, and 270 second- and higher-order textural features. The extracted features are shown in the Supplementary Methods.

### Radiomics imaging biomarker development and validation

For feature selection, the mRMR method was first used to eliminate the redundant and irrelevant features, and then, the LASSO logistic regression method was used to select the most predictive features of the NLR in the training cohort. The dataset was resampled, and the parameters were determined using the expected generalization error estimated from 5-fold cross-validation. The RS was then developed via a linear combination of the selected features weighted by their respective coefficients in the training cohort. The cut-off values of the NLR were created using the tertiles of the RS in the training cohort. The patients were divided into three groups (RS-High, RS-Middle, and RS-Low) based on the tertiles. The group classification details are listed in the Supplementary Methods.

Receiver operating characteristic (ROC) curve analysis was used to evaluate the predictive power of the RS in distinguishing NLR status. The AUCs were used to compare the performance of the radiomics imaging biomarker in each cohort.

### Evaluation of prognosis and anti-PD-1 immunotherapy response

For patients without immunotherapy, Kaplan–Meier curves were used to assess the DFS and OS of the defined RS groups to evaluate the prognostic value of the radiomics imaging biomarker. The log-rank test was used to statistically compare DFS and OS. Univariate and multivariate Cox regression analyses of RS and other clinicopathological variables were performed to select candidate predictors of survival. Moreover, the statistically significant variables (*P* < 0.05) in multivariate Cox regression analyses were incorporated into the nomogram for predicting DFS and OS to improve the predictive power of the radiomics imaging biomarker. Harrell’s concordance index (C-index) was used to assess the discrimination performance of the prognostic nomogram and the radiomics imaging biomarker.

For patients who received immunotherapy, the proportions of different clinical responses were compared based on different RS groups to evaluate the association between the radiomics imaging biomarker and the response to anti-PD-1 immunotherapy. The clinical responses were defined as complete response (CR), partial response (PR), stable disease (SD), or progressive disease (PD) (evaluated at 3 months and 6 months) using RECIST version 1.1^50^. Moreover, PFS and OS were compared for further evaluation.

### Statistical analysis

SPSS version 22.0 (IBM), GraphPad Prism 8, and R version 4.0.2 (http://www.r-project.org) were used for all statistical analyses. A two-tailed *t*-test (unpaired) was used to analyze continuous variable values. The *χ*^2^ test and Fisher’s exact test were used to analyze categorical variables. Univariate and multivariate Cox regression analyses were used to assess the ability of the variables to predict survival. The detailed algorithms used for statistical analysis are described in the Supplementary Methods. A two-sided *P* < 0.05 was considered statistically significant.

### Reporting summary

Further information on research design is available in the Nature Research Reporting Summary linked to this article.

## Supplementary information

Supplementary Information

Reporting summary

## Data Availability

The source data underlying Figs. 2–5, Supplementary Figs. 1–13, and Supplementary Table 10 is provided as a Source data file. The CT imaging data and clinical information, analyzed during the current study are not publicly available for patient privacy purposes. Source data are provided with this paper.

## Source data

Source data

## Code availability

Associated codes to process and analyze data are available on GitHub [https://github.com/Weicai-Huang/Weicai-Huang/tree/main]. Source code has also been placed on the Zenodo platform [10.5281/zenodo.6979909]^54^.
