# Supplementary material for: Noninvasive imaging of the tumor immune microenvironment correlates with response to immunotherapy in gastric cancer
Source: Nat Commun. 2022 Aug 30;13:5095. doi: 10.1038/s41467-022-32816-w (PMC9427761; doi:10.1038/s41467-022-32816-w)
Supplement: Supplementary file 1 — Supplementary Information [file 41467_2022_32816_MOESM1_ESM.pdf]

SUPPLEMENTARY INFORMATION

Noninvasive imaging of the tumor immune microenvironment correlates with response to immunotherapy in gastric cancer

Huang et al.

Supplementary Materials

- **Supplementary Results**
- **Supplementary Methods**
- **Supplementary Figures**
- **Supplementary Tables**
- **Supplementary Reference**

Supplementary Results

Development and validation of radiomics image biomarker

The calculation formula of the RS: 0.02659177* peri-GLSZM_ZSV +0.04131057* peri-GLRLM_RLV +0.05502846* peri-NGTDM_Busyness +0.03061276* intro-NGTDM_Busyness_1.0 +-0.2586982* intro-NGTDM_Strength_1.0 +0.05465974* peri-GLRLM_LRHGE_2.5 +-0.06194509* intro-GLCM_ClusterProminence_1.0 +0.16400638* peri-GLCM_Correlation_1.0 +0.07268216* intro-GLRLM_GLN +0.33539722* peri-perimeter

In this study, the optimum cutoff values of RS to predict NLR status of TIME were created by the tertiles of the RS in the training cohort. According to the values in the training cohort, when RS >= 0.186, patients were divided into RS-High group, and when RS < -0.354, patients were divided into RS-Low group. While when RS >= -0.354 but < 0.186, patients were divided into RS-Middle group.

Supplementary Methods

Patients

In this study, we retrospectively reviewed data for 2272 patients with gastric cancer from three medical centers. For predicting NLR and survival, the patient inclusion criteria were: histologically confirmed gastric adenocarcinoma; standard unenhanced and contrast-enhanced abdominal CT imaging performed <30 days before surgical resection; lymphadenectomy performed and >15 lymph nodes harvested; no preoperative chemotherapy; and complete information about clinicopathological characteristics and follow-up data available. In this study, standard positive threshold

values of CEA and CA19-9 (CEA ≥ 5 ng/mL and CA19-9 ≥ 37 U/mL) were used to divide patients into elevated group and normal group of CEA and CA19-9. We excluded those patients who had other synchronous malignant neoplasms or had received previous anticancer treatment; or if the tumor lesions could not be identified on CT images. For evaluating the response to immunotherapy and clinical outcomes of immunotherapy, the patient inclusion criteria were: histologically confirmed gastric adenocarcinoma; standard unenhanced and contrast-enhanced abdominal CT imaging of primary tumor performed before immunotherapy treatment. Patients were excluded if the clinical response could not be evaluated.

Immunohistochemistry staining and definition of NLR status

In this study, neutrophils and lymphocytes at the center of tumor and invasive margin were stained. Formalin-fixed paraffin-embedded (FFPE) samples were processed for IHC staining as previously described[1-3]. The samples were incubated with antibodies against human CD8 (cytotoxic T lymphocytes; NeoMarker, clone SP16, catalog: MA1-39566) and CD66b (neutrophils; BD Pharmingen, clone G10F5, catalog: 561649), following the staining in an EnVision System (Dako) (Supplementary Table 16). Every staining run contained a slide treated with phosphate buffer saline (PBS) buffer in place of the primary antibody as a negative control. Every staining run contained a slide of positive control. Prior to staining, sections were blocked with endogenous peroxidase (prepared in 1% H_2O_2/methanol solution) for 10 minutes and then microwaved for 30 minutes in 10 mM citrate buffer, pH 6.0. The sections were blocked using 10% normal

rabbit serum for 30 minutes. Furthermore, all slides were stained with the same concentrations of primary antibody for each antibody and incubated with monoclonal primary antibody overnight at 4 ℃, followed by incubation with an amplification system with a labeled polymer/HRP (EnVision™, DakoCytomation, Denmark) at 37℃ for 30 minutes. The sections were developed with 0.05% 3, 3´-diaminobenzidine tetrahydrochloride (DAB) and counterstained with modified Harris hematoxylin. And all slides were stained with DAB dyeing for the same time for each antibody (Supplementary Table 16). Two pathologists (T.L. and S.X. with 5 to 10 years of experience) who were blinded to clinical outcomes independently scored all samples. A third pathologist was consulted when a difference of opinion arose between the 2 primary pathologists. At low power (100), the tissue sections were screened using an inverted research microscope (model DM IRB; Leica, Germeny), and the 5 most representative fields were selected. Thereafter, to evaluate the density of stained immune cells, the 2 respective areas of invasive margin and center of tumor were measured at 200 magnification. The nucleated stained cells in each area were quantified and expressed as the number of cells per field.

We evaluated the NLR (the neutrophil count/the lymphocyte count) in invasive margin (peritumoral area) and center of tumor (intratumoral area) respectively. According to different status of NLR in intratumoral and peritumoral area, the NLR status of TIME were finally classified into three types: NLR High (NLR-H: NLR >=1 both in intratumoral and peritumoral tissue); NLR Mix (NLR-M: NLR >=1 in intratumoral tissue and NLR <1 in peritumoral tissue, or NLR <1 in intratumoral tissue and NLR >=1

in peritumoral tissue); NLR Low (NLR-L: NLR <1 in both intratumoral and peritumoral tissue).

Because IHC data was not available for patients in internal validation cohort 2 and external validation cohort 2, these datasets were used to validate the radiomics image biomarker for the prognostic value, and were not used to evaluate the RS prediction of NLR.

Image acquisition, processing and feature extraction

For patients who were treated without immunotherapy, portal venous-phase CT images taken before the surgery treatment were obtained from the picture archiving and communication system (PACS, Carestream Canada). For those patients who received immunotherapy, portal venous-phase CT images of primary tumor were obtained before the immunotherapy. All patients underwent contrast-enhanced abdominal CT using the multidetector row CT (MDCT) systems (GE Lightspeed 16, GE Healthcare Milwaukee, WI; 64-section LightSpeed VCT, GE Medical Systems, Milwaukee, WI; USA). Following intravenous contrast administration, arterial and portal venous-phase contrast-enhanced CT scans were performed after delays of 28 s and 60 s, respectively. Iodinated contrast material in the amount of 90 - 100 ml (Ultravist 370, Bayer Schering Pharma, Berlin, Germany) was injected at a rate of 3.0 or 3.5 ml/s with a pump injector (Ulrich CT Plus 150, Ulrich Medical, Ulm, Germany). The CT acquisition protocols were as follows: 120 kV; 150-190 mAs; 0.5- or 0.4-second rotation time. Contrast-enhanced CT was reconstructed with a field of view, 350×350 mm; data matrix,

512×512; in-plane spatial resolution 0.607-0.751 mm; axial slice thickness 5.0 mm for 98% patients with a range of 1.25-7.5 mm.

Because of a coarse resolution in z-axis compared with in-plane resolution, different axial slice thickness may lead to a bias in the synthesized 3D image and potentially higher inter-rater variability, which may adversely affect model performance. Hence, we focused on the most representative image slice, i.e., largest tumor section in the axial plane. Two radiologists (CC and QY who have 12 and 11 years of clinical experience in abdominal CT interpretation respectively) manually segmented the CT images using the ITK-SNAP software (www.itksnap.org). The CT number (i.e., Hounsfield units) was normalized with the soft tissue window of [-350, 450] HU. Both radiologists were blinded to the clinical and histopathological data but were aware that the patients had gastric cancer. All tumor contours were delineated by the two radiologists in consensus, and any discrepancy was resolved by a third radiologist (Y.X. with 32 years of experience in abdominal CT interpretation). In general, the image processing was followed The Image Biomarker Standardization Initiative (IBSI) guidelines (Supplementary Table 17).

In this study, a total of 584 quantitative features (292 in the peritumoral area and 292 in the intratumornal area, respectively) of each ROI were extracted. The image features included 14 first-order intensity features, 8 shape features, and 270 second- and higher-order textural features. In this work, we investigated four types of texture features on the basis of gray-level co-occurrence matrices (GLCM), gray-level run length matrix (GLRLM), gray-level size zone matrix (GLSZM), and neighborhood gray-tone

difference matrix wavelet decompositions (NGTDM). A Laplacian of Gaussian spatial band-pass filter ($\nabla^2 G$) was used to derive image features at different spatial scales by turning the filter parameter between 1.0 and 2.5 (1.0, 1.5, 2.0, 2.5). All the image features were implemented and computed using an open-source radiomics analysis package in the MATLAB platform (https://github.com/mvallieres/radiomics). The source code used in this work is publicly available at https://github.com/yumingjiang/GC_RADIOMICS-.git.

Classification of RS groups

In this study, the range of the RS in the training is -1.671 to 1.936, and the cutoff value of the bottom and middle tertiles is -0.354, while the cutoff value of the middle and top tertiles is 0.186. Therefore, patients with RS lower than -0.354 were divided into RS-Low group, and patients with RS higher than or equal to 0.186 were divided into RS-High group. While those patients with RS higher than or equal to -0.354 but lower than 0.186 were divided into RS-Middle group.

Statistical analysis

Redundant and irrelevant features elimination was performed by using the mRMR algorithm in “mRMRe” package; Predictive features selections were performed by using the LASSO logistic regression algorithm in “glmnet” package; ROC curves were plotted by using the “pROC” packages; Nomogram were constructed by using the “rms” package; Survival curves were plotted by using the “survminer” packages.

Supplementary Figures

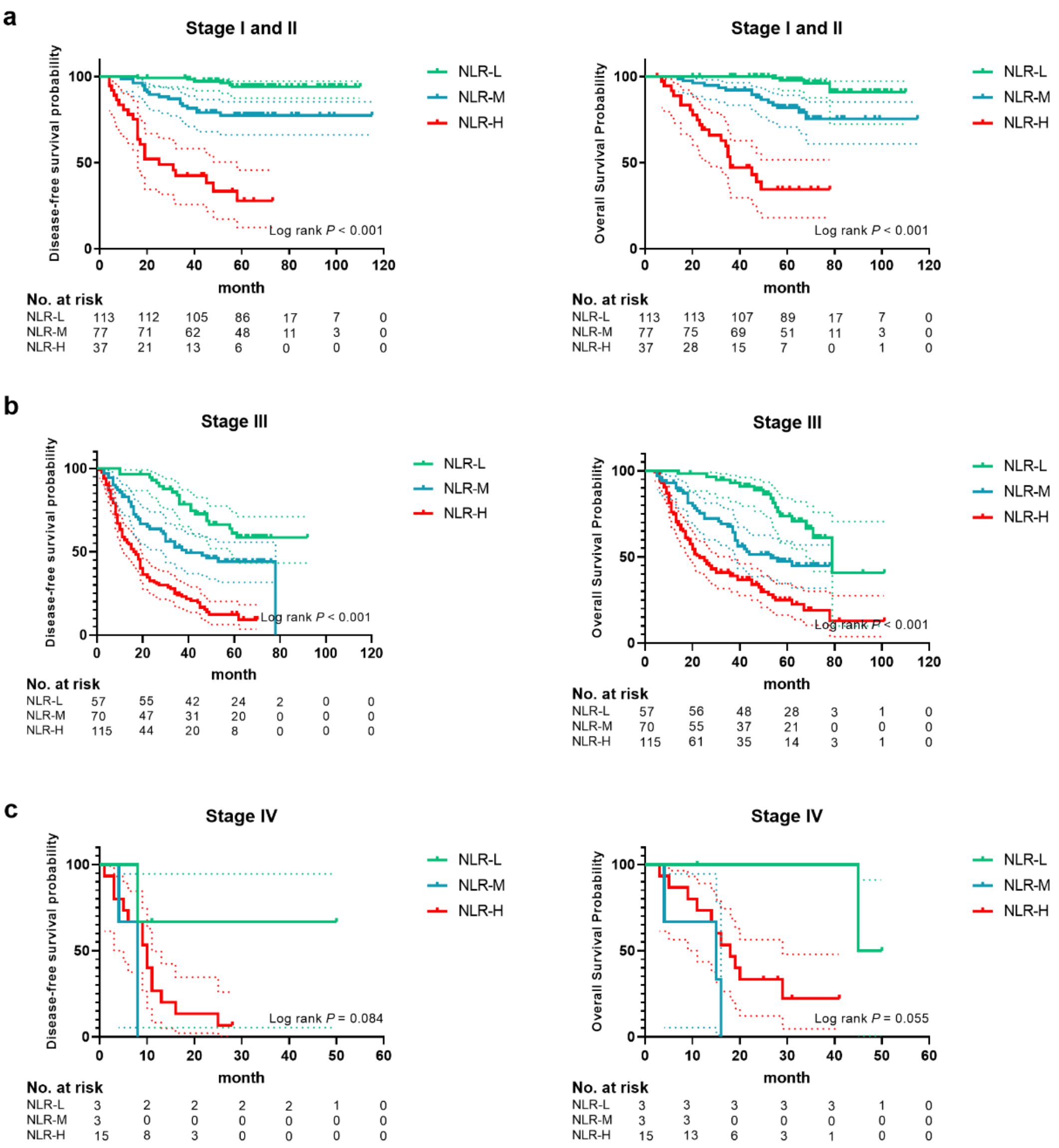

Supplementary Fig. 1. Kaplan-Meier survival analysis of disease-free survival and overall survival according to the NLR status of TIME in different stage of the training and validation cohort. a: Patients in stage I and II (n = 227), b: Patients in stage III (n = 242), c: Patients in stage IV (n = 21). NLR: neutrophils-to-lymphocytes ratio, NLR-L: NLR-Low group, NLR-M: NLR-Mix group, NLR-H: NLR-High group, comparisons of the above progression survival curves were performed with a two-sided log-rank test. Dashed lines around the survival curves represent 95% confidence intervals. Source data are provided as a Source Data file.

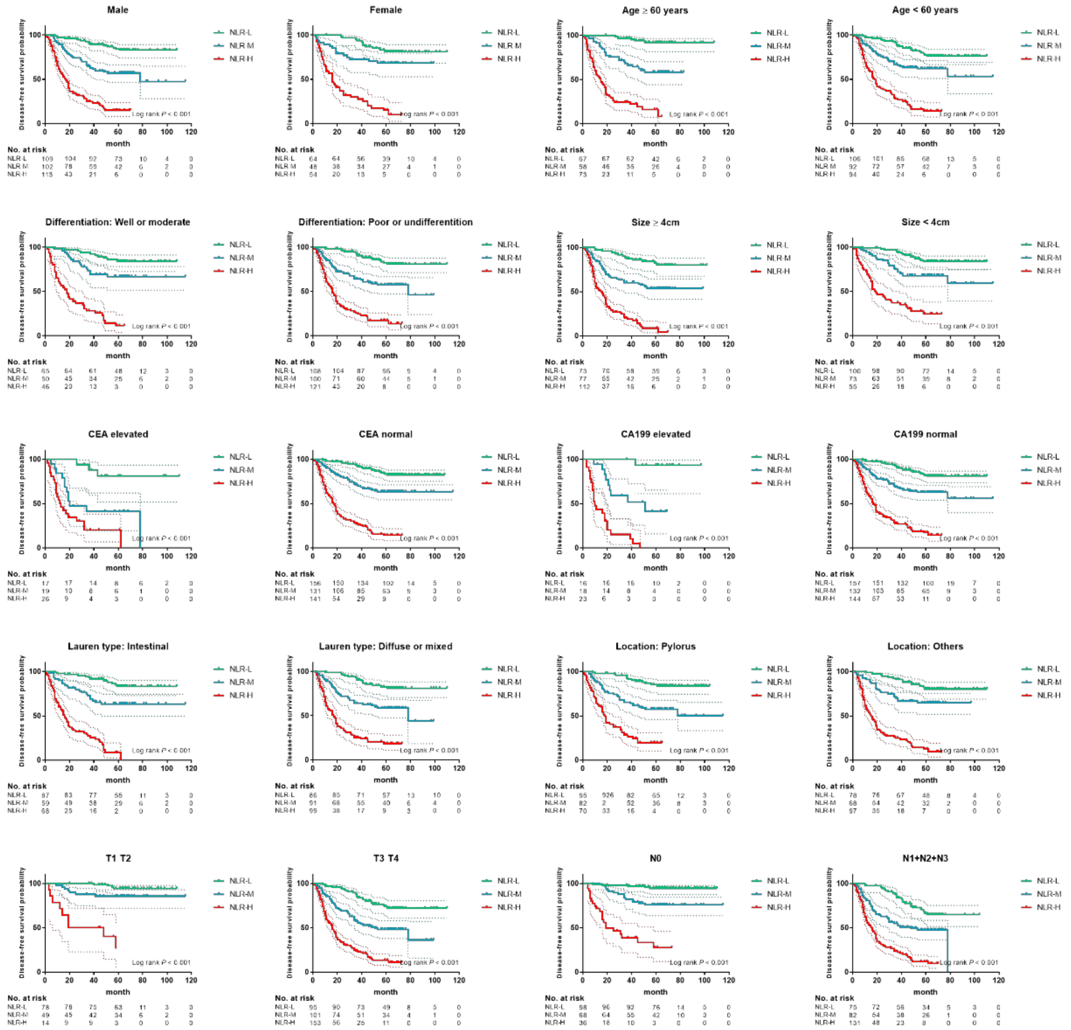

Supplementary Fig. 2. Kaplan-Meier survival analysis of DFS for patients with NLR status (n = 490) according to the NLR status of TIME stratified by clinicopathological risk factors. NLR: neutrophils-to-lymphocytes ratio, NLR-L: NLR-Low group, NLR-M: NLR-Mix group, NLR-H: NLR-High group, comparisons of the above progression survival curves were performed with a two-sided log-rank test. Dashed lines around the survival curves represent 95% confidence intervals. Source data are provided as a Source Data file.

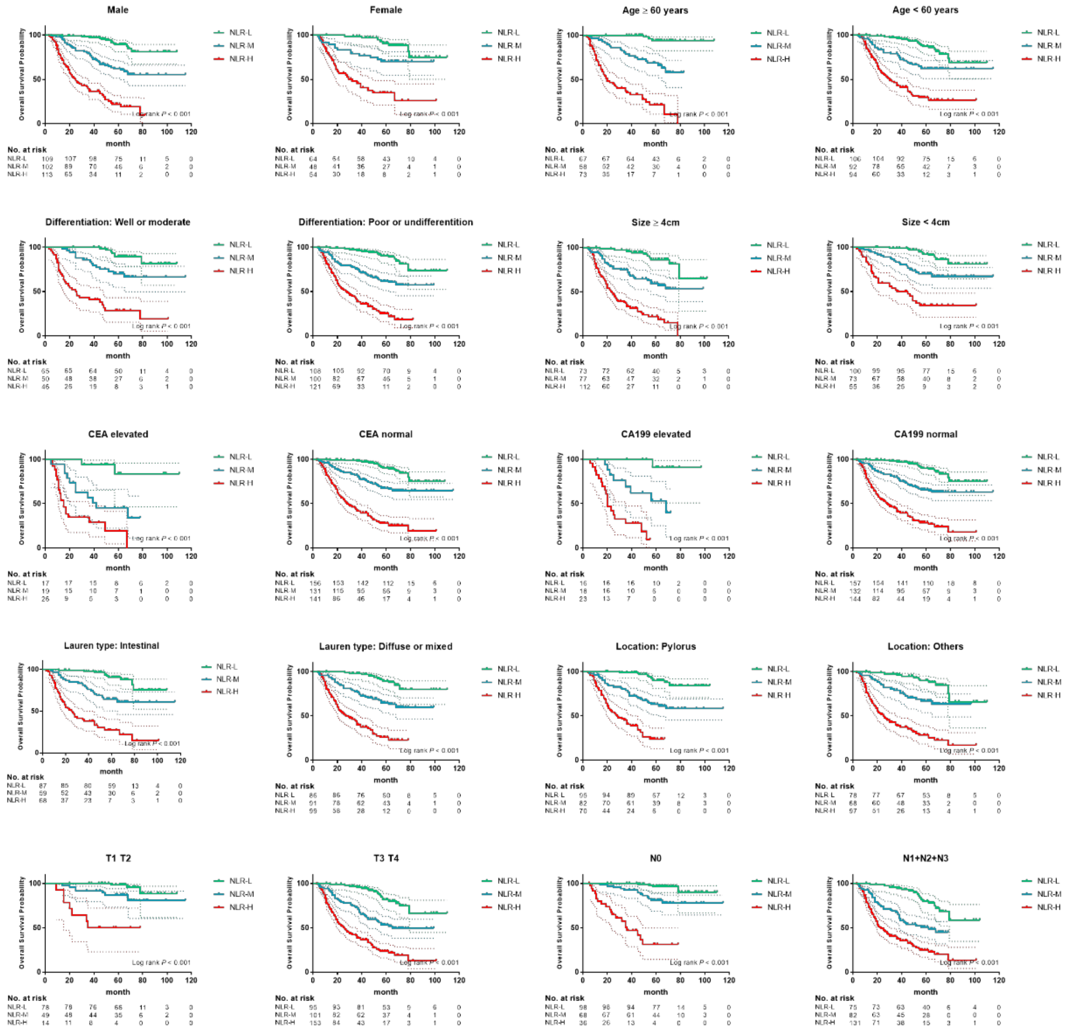

Supplementary Fig. 3. Kaplan-Meier survival analysis of OS for patients with NLR status (n = 490) according to the NLR status of TIME stratified by clinicopathological risk factors. NLR: neutrophils-to-lymphocytes ratio, NLR-L: NLR-Low group, NLR-M: NLR-Mix group, NLR-H: NLR-High group, comparisons of the above progression survival curves were performed with a two-sided log-rank test. Dashed lines around the survival curves represent 95% confidence intervals. Source data are provided as a Source Data file.

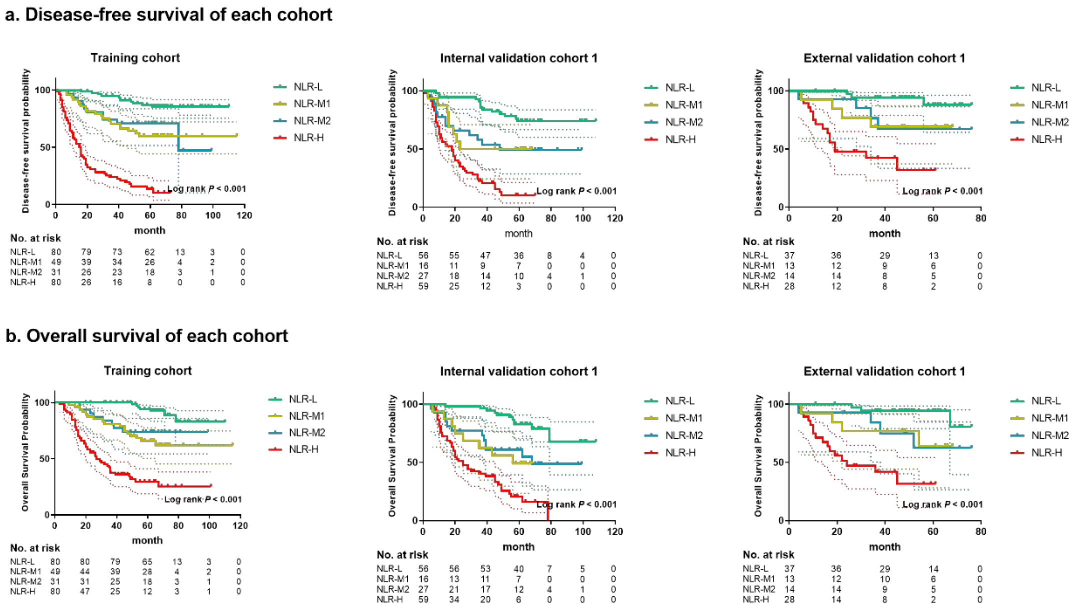

Supplementary Fig. 4. Kaplan-Meier survival analysis of disease-free survival and overall survival according to the NLR status of TIME in the training, internal validation cohort 1 and external validation cohort 1. a: disease-free survival, b: overall survival. Training cohort: n=240, internal validation cohort 1: n=158, external validation cohort 1: n=92, NLR: neutrophils-to-lymphocytes ratio, NLR-L: NLR-Low group, NLR-M1: NLR-Mix 1 group, NLR-M2 group: NLR-Mix 2 group, NLR-H: NLR-High group, comparisons of the above progression survival curves were performed with a two-sided log-rank test. Dashed lines around the survival curves represent 95% confidence intervals. Source data are provided as a Source Data file.

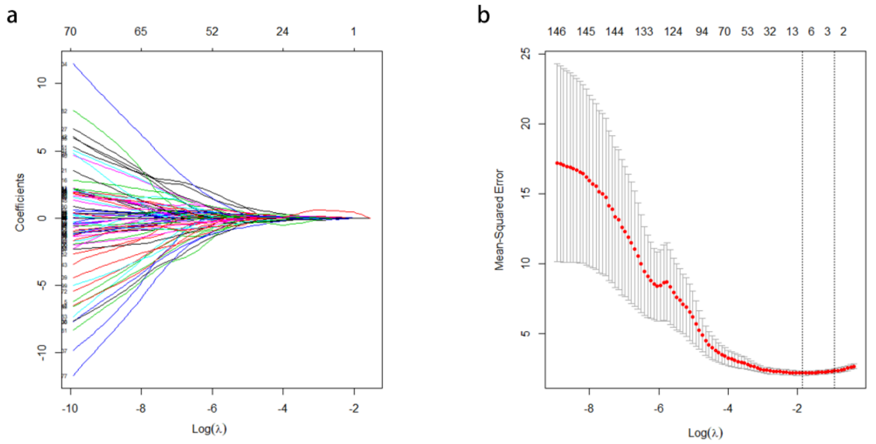

Supplementary Fig. 5. Texture feature selection using the least absolute shrinkage and selection operator (LASSO) logistic regression model. a: LASSO coefficient profiles of the image texture feature. A coefficient profile plot was produced against the

log (λ) sequence, b: tuning parameter (λ) selection in the LASSO model used 5-fold cross-validation via minimum criteria. Number of features selected corresponding to each lambda are given above the plot. Solid vertical lines represent the mean squared error ± SE, the centre red points of the solid vertical lines represent the mean squared error under different λ values, and the error bars indicate the SE. Dotted vertical lines were drawn at the optimal values by using the minimum criteria and 1 standard error of the minimum criteria (the 1-SE criteria). A λ value of 0.1560146, with log (λ) of -1.857805 was chosen (minimum criteria) according to 5-fold cross-validation. Ten features were selected. SE: standard error. Source data are provided as a Source Data file.

a. Training cohort

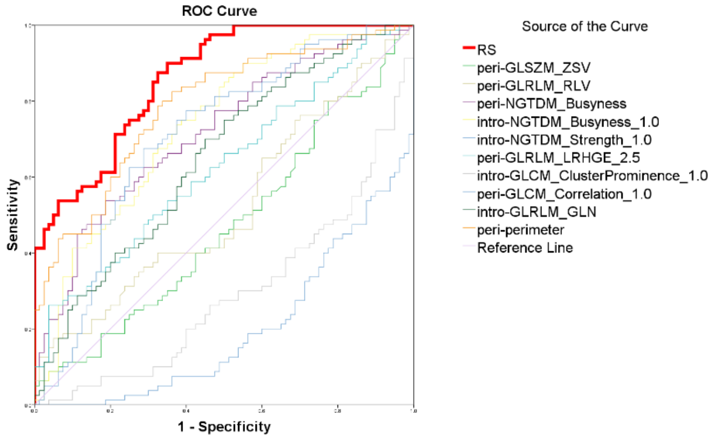

b. Internal validation cohort 1

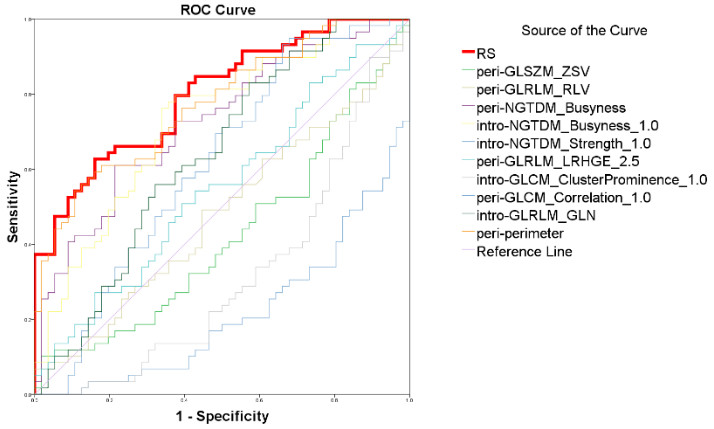

c. External validation cohort 1

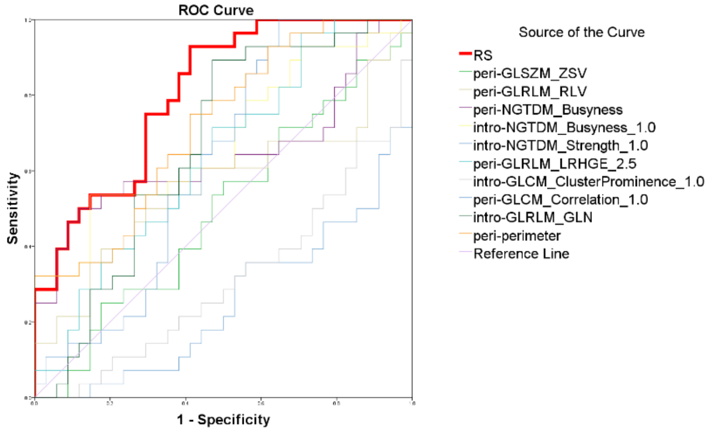

Supplementary Fig. 6. ROC curves of RS and the ten selected features in predicting NLR status in the training cohort, internal validation cohort 1, and external validation 1. RS: radiomics score. Source data are provided as a Source Data file.

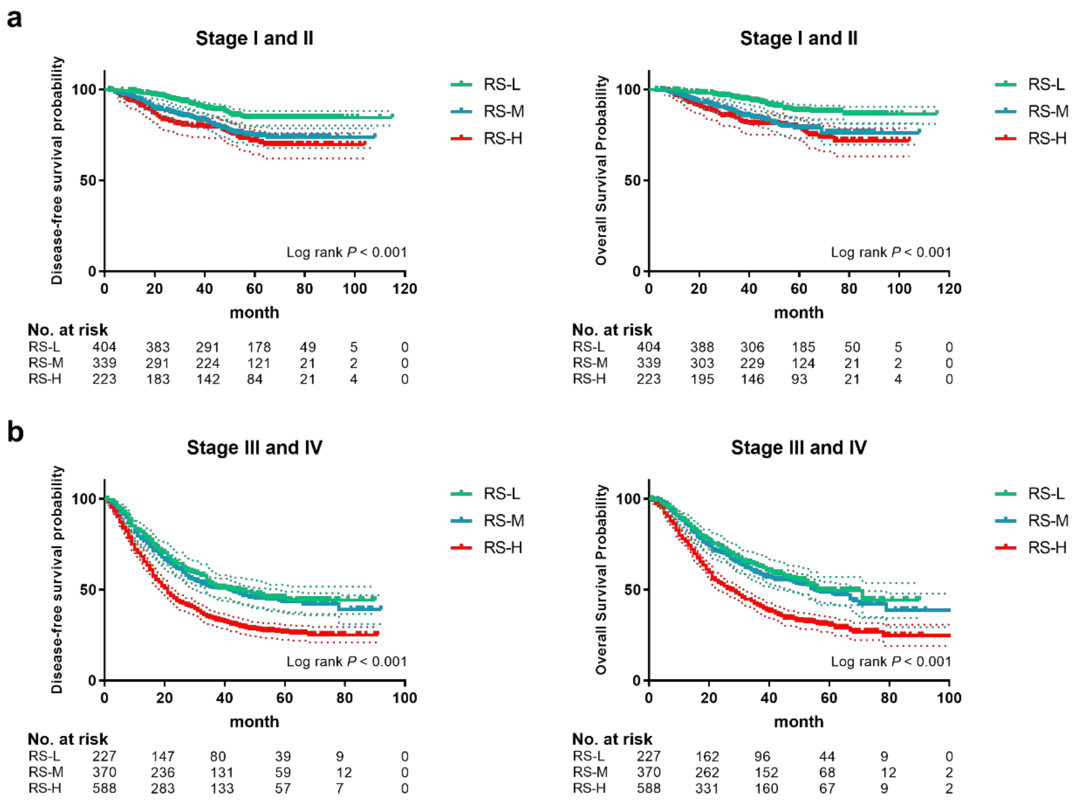

Supplementary Fig. 7. Kaplan-Meier survival analysis of disease-free survival and overall survival according to the RS groups in patients with different stage of the training and validation cohorts. a: Stage I and II (n = 966), b: Stage III and IV (n = 1185). RS-L: RS-Low group, RS-M: RS-Middle group, RS-H: RS-High group, comparisons of the above progression survival curves were performed with a two-sided log-rank test. Dashed lines around the survival curves represent 95% confidence intervals. Source data are provided as a Source Data file.

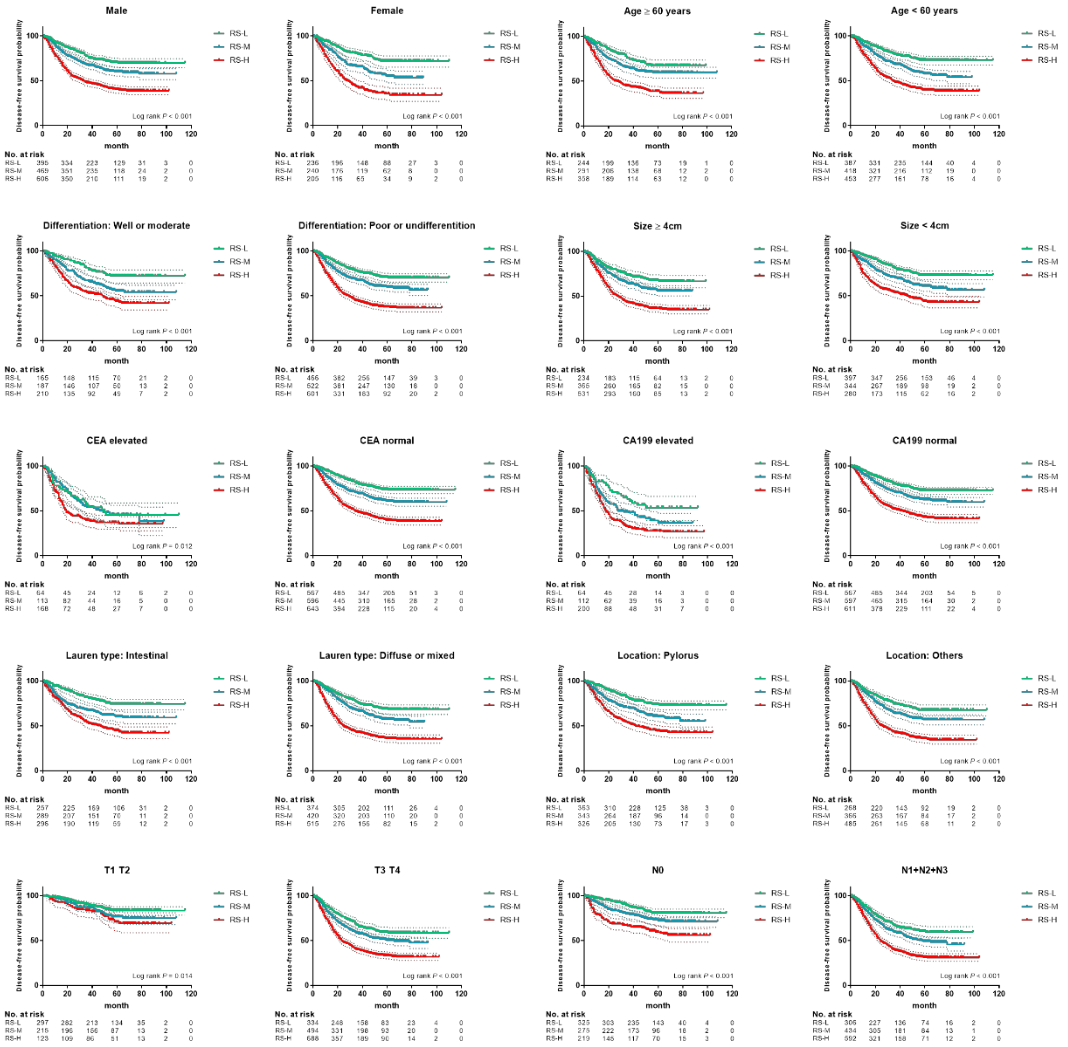

Supplementary Fig. 8. Kaplan-Meier survival analysis of DFS for all the 2,151 patients according to the RS groups stratified by clinicopathological risk factors. RS-L: RS-Low group, RS-M: RS-Middle group, RS-H: RS-High group, comparisons of the above progression survival curves were performed with a two-sided log-rank test. Dashed lines around the survival curves represent 95% confidence intervals. Source data are provided as a Source Data file.

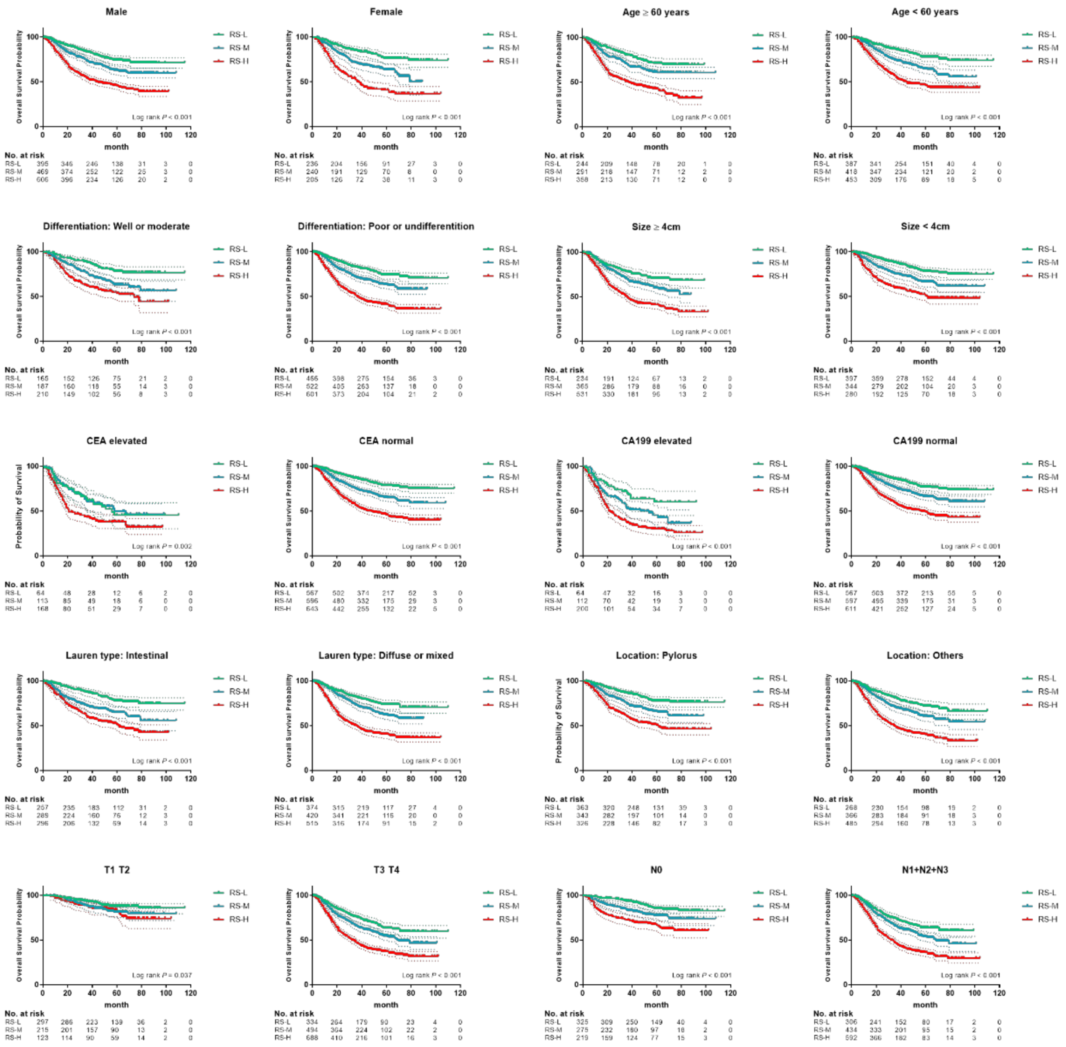

Supplementary Fig. 9. Kaplan-Meier survival analysis of OS for all the 2,151 patients according to the RS groups stratified by clinicopathological risk factors. RS-L: RS-Low group, RS-M: RS-Middle group, RS-H: RS-High group, comparisons of the above progression survival curves were performed with a two-sided log-rank test. Dashed lines around the survival curves represent 95% confidence intervals. Source data are provided as a Source Data file.

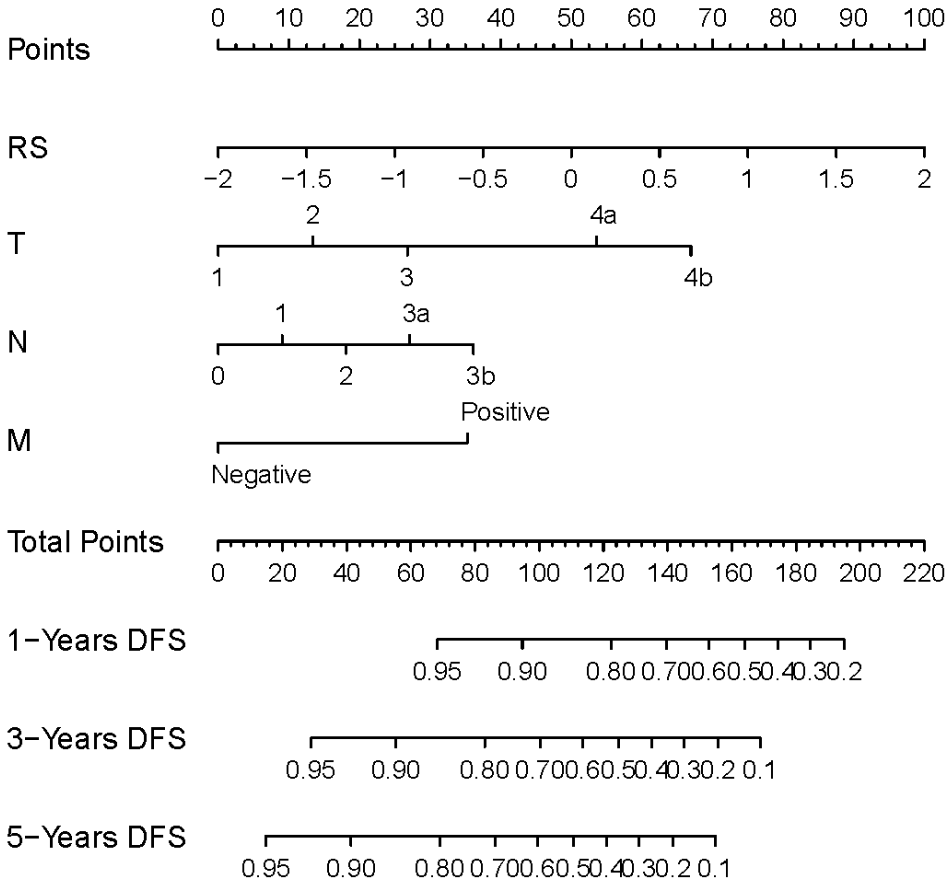

Supplementary Fig. 10. Integrated nomograms to predict 1-, 3-, 5- year DFS for patients with gastric cancer. To determine how many points toward the probability of DFS, the patient receives for his or her RS, locate the patient's RS on the RS axis, draw a line straight upward to the point axis, repeat this process for each variable, sum the points achieved for each of the risk factors, locate the final sum on the Total Point axis, and draw a line straight down to find the patient's probability of DFS. Source data are provided as a Source Data file.

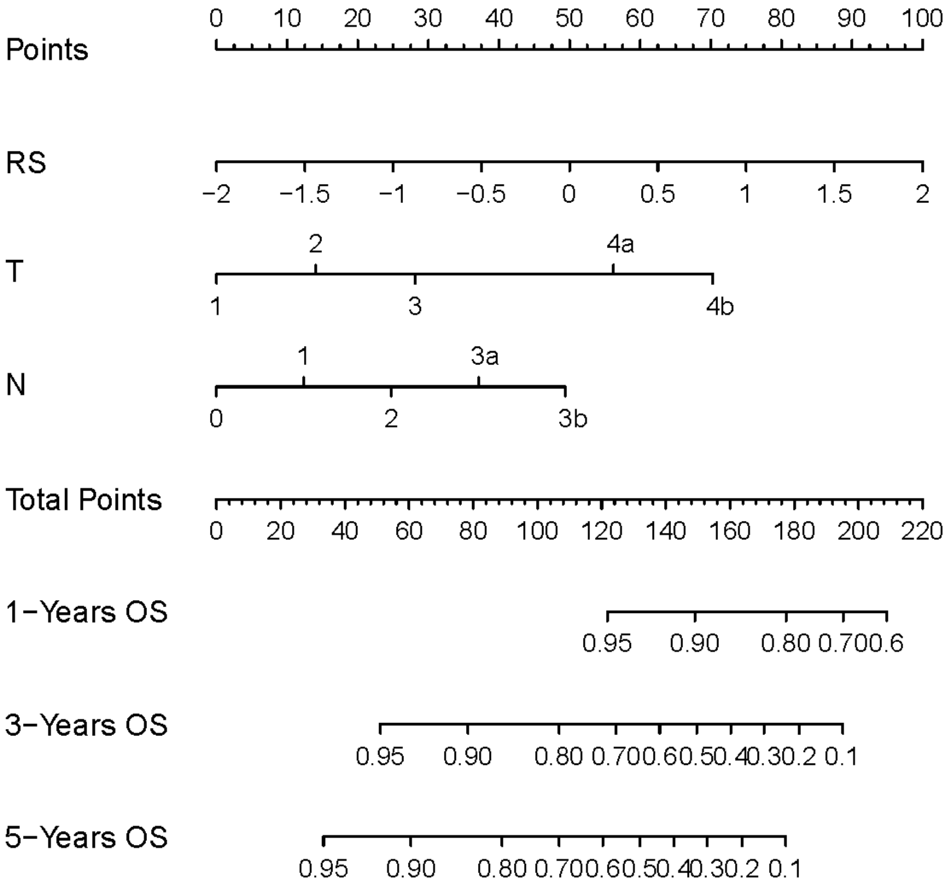

Supplementary Fig. 11. Integrated nomograms to predict 1-, 3-, 5- year OS for patients with gastric cancer. To determine how many points toward the probability of OS, the patient receives for his or her RS, locate the patient's RS on the RS axis, draw a line straight upward to the point axis, repeat this process for each variable, sum the points achieved for each of the risk factors, locate the final sum on the Total Point axis, and draw a line straight down to find the patient's probability of OS. Source data are provided as a Source Data file.

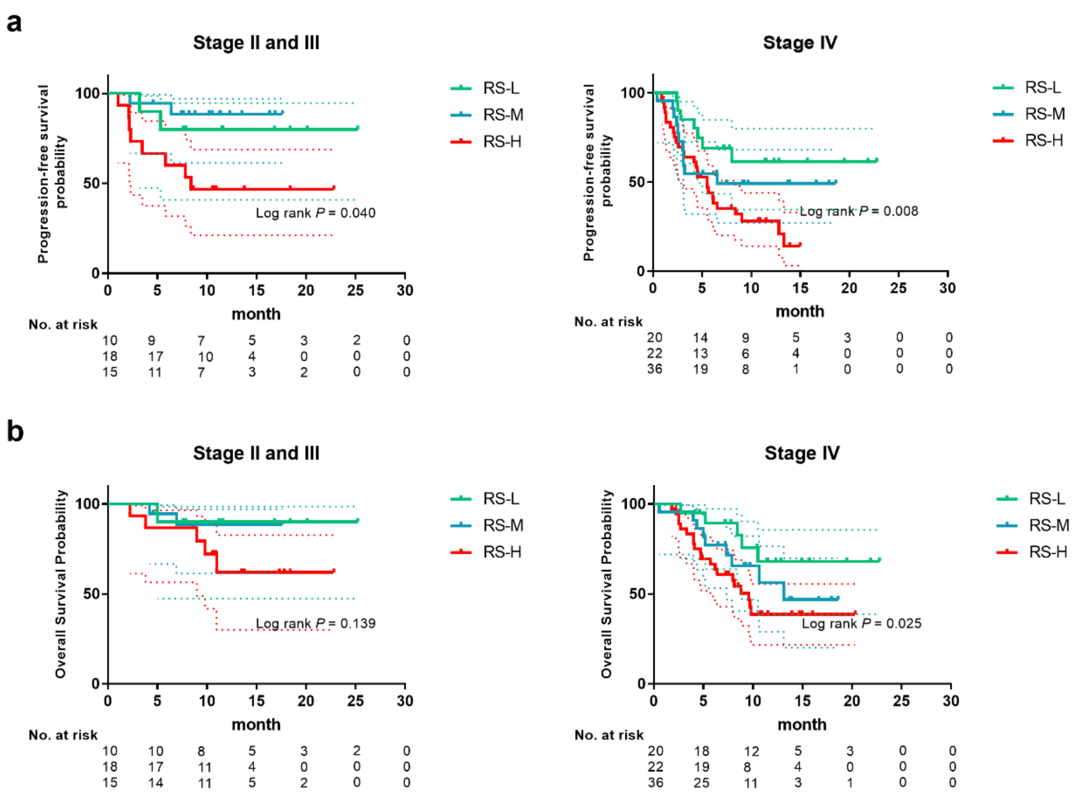

Supplementary Fig. 12. Kaplan-Meier survival analysis of PFS and OS for patients who received immunotherapy according to the RS groups stratified by stage of disease. a: disease-free survival. b: overall survival. RS-L: RS-Low group, RS-M: RS-Middle group, RS-H: RS-High group, comparisons of the above progression survival curves were performed with a two-sided log-rank test. Dashed lines around the survival curves represent 95% confidence intervals. Source data are provided as a Source Data file.

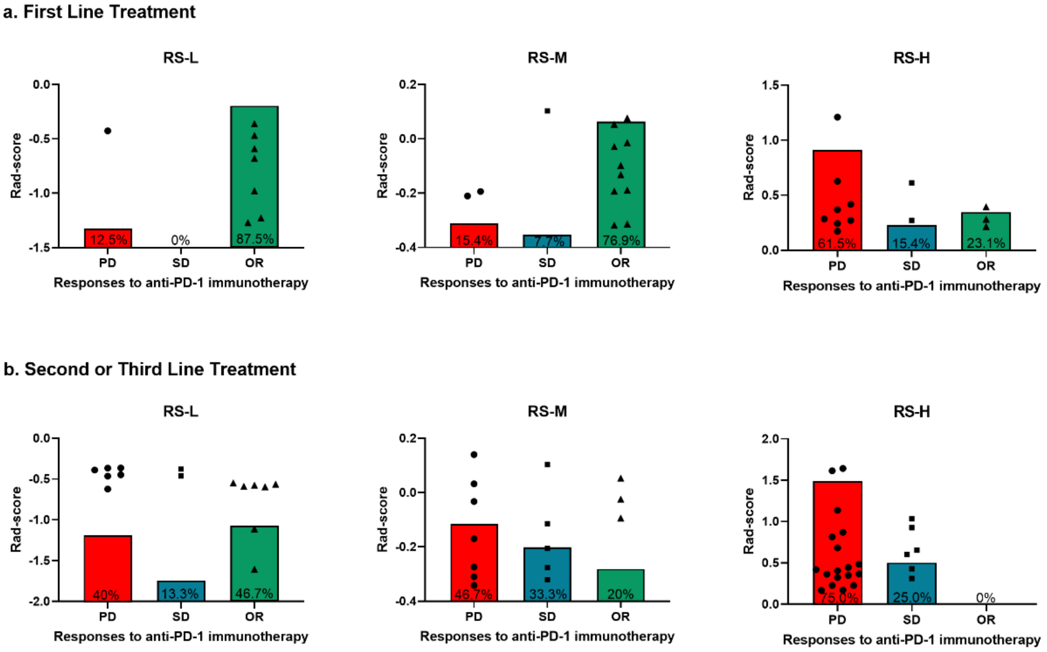

Supplementary Fig. 13. Evaluation of the response to anti-PD-1 immunotherapy in subgroup analysis, according to the RS groups. a: The Radiomics scores of patients and proportions of different responses to anti-PD-1 immunotherapy in patients who received immunotherapy as the first line treatment, b: the Radiomics scores of patients and proportions of different responses to anti-PD-1 immunotherapy in patients who received immunotherapy as the second or the third line treatment. RS-L: RS-Low group, RS-M: RS-Middle group, RS-H: RS-High group. Source data are provided as a Source Data file.

Supplementary Tables

Supplementary Table 1. Characteristics of patients with GC in anti-PD-1 immunotherapy cohorts.

Variables	SMU cohort n = 88		GPHCM cohort n = 33	
	n	%	n	%
Gender				
Male	51	58	12	36.4
Female	37	42	21	63.6
Age (years), median (interquartile range)	54 (46-65）		60 (49-66)	
Stage				
II	5	5.7	0	0
III	30	34.1	8	24.2
IV	53	60.2	25	75.8
Treatment line (Immunotherapy)				
First Line	34	38.6	0	0
Second line	28	31.8	21	63.6
Third line	26	29.6	12	36.4
Treatment response				
CR	7	8.0	1	3.0
PR	23	26.1	7	21.2
SD	16	18.2	10	30.3
PD	42	47.7	15	45.5

CR: complete response, PR: partial response, SD: stable disease, PD: progressive disease.

Supplementary Table 2. Clinical characteristics of patients according to the NLR status in the training cohort.

Variables	Training cohort (n = 240)			
	NLR-L(%)	NLR-M(%)	NLR-H(%)	*P*
Gender				0.882[b]
Male	49(61.2)	52(65.0)	50(62.5)	
Female	31(38.8)	28(35.0)	30(37.5)	
Size				0.026[b]
≥4cm	32(40.0)	39(48.8)	49(61.2)	
<4cm	48(60.0)	41(51.2)	31(38.8)	
Age				0.8[b]
≥60	31(38.8)	34(52.5)	35(43.8)	
<60	49(61.2)	46(57.5)	45(56.2)	
Differentiation				0.365[b]
Well	11(13.8)	9(11.3)	7(8.8)	
Moderate	25(31.2)	18(22.5)	17(21.2)	
Poor or undifferentiation	44(55.0)	53(66.3)	56(70.0)	
Location				0.462[a]
Cardia	15(18.8)	16(20.0)	19(23.8)	
Body	15(18.8)	14(17.5)	18(22.4)	
Antrum	45(56.2)	48(60.0)	36(45.0)	
Whole	5(6.2)	2(2.5)	7(8.8)	
Lauren type				0.727[b]
Intestinal type	39(48.8)	37(46.2)	34(42.5)	
Diffuse or mixed type	41(51.2)	43(53.8)	46(57.5)	
CEA				0.441[b]
Elevated	6(7.5)	9(11.2)	11(13.8)	
Normal	74(92.5)	71(88.8)	69(86.2)	
CA19-9				0.022[b]
Elevated	8(10.0)	6(7.5)	17(21.2)	
Normal	72(90)	74(92.5)	63(78.8)	
Depth of invasion				<0.001[a]
T1	31(38.8)	22(27.4)	2(2.5)	
T2	8(10.0)	9(11.3)	4(5.0)	
T3	10(12.4)	12(15.0)	7(8.8)	
T4a	28(35.0)	32(40.0)	50(62.5)	
T4b	3(3.8)	5(6.3)	17(21.2)	
Lymph node metastasis				<0.001[a]
N0	50(62.4)	42(52.4)	18(22.4)	
N1	11(13.8)	17(21.3)	16(20.0)	
N2	7(8.8)	6(7.5)	9(11.3)	
N3a	8(10.0)	6(7.5)	21(26.3)	
N3b	4(5.0)	9(11.3)	16(20.0)	
Distant metastasis				0.018[a]
Yes	0(0)	1(1.3)	6(7.5)	
No	80(100)	79(98.7)	74(92.5)	
TNM stage				<0.001[a]
I	36(45.0)	27(33.8)	2(2.5)	

II	19(23.8)	19(23.8)	20(25.0)
III	25(31.2)	33(41.2)	52(65.0)
IV	0(0)	1(1.2)	6(7.5)

Variables are in n (%). [a]: *P* values are two-tailed from Fisher's exact test, [b]: *P* values are two-tailed from $\chi 2$ test.

Supplementary Table 3. Clinical characteristics of patients according to the NLR status in the internal validation cohort 1.

Variables	Internal validation cohort 1 (n = 158)			
	NLR-L(%)	NLR-M(%)	NLR-H(%)	*P*
Gender				0.822[b]
Male	38(67.9)	31(72.1)	43(72.9)	
Female	18(32.1)	12(27.9)	16(27.1)	
Size				0.005[b]
≥4cm	22(39.3)	23(53.5)	41(69.5)	
<4cm	34(60.7)	20(46.5)	18(30.5)	
Age				0.816[b]
≥60	18(32.1)	14(32.6)	22(37.3)	
<60	38(67.9)	29(67.4)	37(62.7)	
Differentiation				0.746[a]
Well	5(8.9)	1(4.7)	3(5.1)	
Moderate	16(28.6)	14(32.5)	14(23.7)	
Poor or undifferentiation	35(62.5)	27(62.8)	42(71.2)	
Location				0.425[a]
Cardia	13(23.2)	8(18.5)	17(28.8)	
Body	7(12.5)	7(16.3)	14(23.7)	
Antrum	33(58.9)	26(60.5)	24(40.7)	
Whole	3(5.4)	2(4.7)	4(6.8)	
Lauren type				0.031[b]
Intestinal type	34(60.7)	15(34.9)	26(44.1)	
Diffuse or mixed type	22(39.3)	28(65.1)	33(55.9)	
CEA				0.861[b]
Elevated	6(10.7)	6(14.0)	8(13.6)	
Normal	50(89.3)	37(86.0)	51(86.4)	
CA19-9				0.228[a]
Elevated	4(7.1)	7(16.3)	4(6.8)	
Normal	52(92.9)	36(83.7)	55(93.2)	
Depth of invasion				<0.001[a]
T1	17(30.4)	8(18.6)	2(3.4)	
T2	6(10.7)	5(11.6)	3(5.1)	
T3	10(17.9)	6(14.0)	3(5.1)	
T4a	18(32.1)	15(34.9)	35(59.3)	
T4b	5(8.9)	9(20.9)	16(27.1)	
Lymph node metastasis				<0.001[a]
N0	33(58.9)	15(34.9)	10(16.9)	
N1	7(12.5)	10(23.2)	9(15.3)	
N2	12(21.5)	6(14.0)	9(15.3)	
N3a	4(7.1)	7(16.3)	18(30.5)	
N3b	0(0)	5(11.6)	13(22.0)	
Distant metastasis				0.389[a]
Yes	1(1.8)	1(2.3)	4(6.8)	
No	55(98.2)	42(97.7)	55(93.2)	
TNM stage				<0.001[a]
I	21(37.5)	10(23.3)	3(5.1)	

II	15(26.8)	8(18.6)	7(11.9)
III	19(33.9)	24(55.8)	45(76.2)
IV	1(1.8)	1(2.3)	4(6.8)

Variables are in n (%). [a]: *P* values are two-tailed from Fisher's exact test, [b]: *P* values are two-tailed from $\chi 2$ test.

Supplementary Table 4. Clinical characteristics of patients according to the NLR status in the external validation cohort 1.

Variables	External validation cohort 1 (n = 92)			
	NLR-L(%)	NLR-M(%)	NLR-H(%)	*P*
Gender				0.521[b]
Male	22(59.5)	19(70.4)	20(71.4)	
Female	15(40.5)	8(29.6)	8(28.6)	
Size				0.067[b]
≥4cm	19(51.4)	15(55.6)	22(78.6)	
<4cm	18(48.6)	12(44.4)	6(21.4)	
Age				0.326[b]
≥60	18(48.6)	10(37.0)	16(57.1)	
<60	19(51.4)	17(63.0)	12(42.9)	
Differentiation				0.905[a]
Well	1(2.7)	0(0)	0(0)	
Moderate	7(18.9)	7(25.9)	5(17.9)	
Poor or undifferentiation	29(78.4)	20(74.1)	23(82.1)	
Location				0.550[a]
Cardia	10(27.0)	10(37.0)	9(32.1)	
Body	7(18.9)	9(33.3)	7(25.0)	
Antrum	17(45.9)	8(29.7)	10(35.8)	
Whole	3(8.2)	0(0)	2(7.1)	
Lauren type				0.552[b]
Intestinal type	14(37.8)	7(25.9)	8(28.6)	
Diffuse or mixed type	23(62.2)	20(74.1)	20(71.4)	
CEA				0.476[a]
Elevated	5(13.5)	4(14.8)	7(25.0)	
Normal	32(86.5)	23(85.2)	21(75.0)	
CA19-9				0.394[a]
Elevated	4(10.8)	5(18.5)	2(7.1)	
Normal	33(89.2)	22(81.5)	26(92.9)	
Depth of invasion				0.051[a]
T1	8(21.6)	4(14.8)	2(7.1)	
T2	8(21.6)	1(3.7)	1(3.6)	
T3	7(18.9)	5(18.5)	9(32.1)	
T4a	11(29.8)	16(59.3)	11(39.3)	
T4b	3(8.1)	1(3.7)	5(17.9)	
Lymph node metastasis				0.328[a]
N0	15(40.6)	11(40.8)	8(28.6)	
N1	7(18.9)	4(14.8)	3(10.7)	
N2	5(13.5)	3(11.1)	3(10.7)	
N3a	4(10.8)	8(29.6)	7(25.0)	
N3b	6(16.2)	1(3.7)	7(25.0)	
Distant metastasis				0.202[a]
Yes	2(5.4)	1(3.7)	5(17.9)	
No	35(94.6)	26(96.3)	23(82.1)	
TNM stage				0.027[a]

I	11(29.7)	5(18.5)	3(10.7)
II	11(29.7)	8(29.6)	2(7.1)
III	13(35.1)	13(48.2)	18(64.3)
IV	2(5.5)	1(3.7)	5(17.9)

Variables are in n (%), [a]: *P* values are two-tailed from Fisher's exact test, [b]: *P* values are two-tailed from $\chi 2$ test.

Supplementary Table 5. Clinical characteristics of patients according to the RS in the training cohort.

Variables	Training cohort (n = 240)			
	RS-L(%)	RS-M(%)	RS-H(%)	*P*
Gender				0.024[b]
Male	41(51.2)	57(71.3)	53(66.2)	
Female	39(48.8)	23(28.7)	27(33.8)	
Size				<0.001[b]
≥4cm	28(35.0)	38(47.5)	54(67.5)	
<4cm	52(65.0)	42(52.5)	26(32.5)	
Age				0.154[b]
≥60	27(33.8)	34(42.5)	39(48.8)	
<60	53(66.2)	46(57.5)	41(51.2)	
Differentiation				0.044[b]
Well	14(17.5)	11(13.8)	2(2.5)	
Moderate	18(22.5)	20(25.0)	22(27.5)	
Poor or undifferentiation	48(60.0)	49(61.2)	56(70.0)	
Location				0.395[a]
Cardia	11(13.8)	20(25.0)	19(23.8)	
Body	19(23.8)	15(18.8)	13(16.2)	
Antrum	46(57.4)	42(52.4)	41(51.2)	
Whole	4(5.0)	3(3.8)	7(8.8)	
Lauren type				0.439[b]
Intestinal type	41(51.2)	36(45.0)	33(41.3)	
Diffuse or mixed type	39(48.8)	44(55.0)	47(58.7)	
CEA				0.203[b]
Elevated	5(6.3)	9(11.3)	12(15.0)	
Normal	75(93.7)	71(88.7)	68(85.0)	
CA19-9				0.004[b]
Elevated	4(5.0)	9(11.3)	18(22.5)	
Normal	76(95.0)	71(88.7)	62(77.5)	
Depth of invasion				<0.001[a]
T1	41(51.1)	13(16.2)	1(1.2)	
T2	7(8.8)	7(8.8)	7(8.8)	
T3	11(13.8)	10(12.5)	8(10.0)	
T4a	18(22.5)	44(55.0)	48(60.0)	
T4b	3(3.8)	6(7.5)	16(20.0)	
Lymph node metastasis				<0.001[a]
N0	56(70.0)	39(48.8)	15(18.8)	
N1	10(12.4)	12(15.0)	22(27.4)	
N2	3(3.8)	8(10.0)	11(13.8)	
N3a	7(8.8)	12(15.0)	16(20.0)	
N3b	4(5.0)	9(11.2)	16(20.0)	
Distant metastasis				0.073[a]
Yes	0(0)	2(2.5)	5(6.3)	
No	80(100)	78(97.5)	75(93.7)	
TNM stage				<0.001[a]
I	44(55.0)	16(20.0)	5(6.3)	

II	17(21.3)	26(32.5)	15(18.7)
III	19(23.7)	36(45.0)	55(68.7)
IV	0(0)	2(2.5)	5(6.3)

Variables are in n (%), [a]: *P* values are two-tailed from Fisher's exact test, [b]: *P* values are two-tailed from $\chi 2$ test.

Supplementary Table 6. Clinical characteristics of patients according to the RS in the internal validation cohort 1.

Variables	Internal validation cohort 1 (n = 158)			
	RS-L(%)	RS-M(%)	RS-H(%)	*P*
Gender				0.261[b]
Male	25(69.4)	38(64.4)	49(77.8)	
Female	11(30.6)	21(35.6)	14(22.2)	
Size				<0.001[b]
≥4cm	11(30.6)	28(47.5)	47(74.6)	
<4cm	25(69.4)	31(52.5)	16(25.4)	
Age				0.53[b]
≥60	15(41.7)	18(30.5)	21(33.3)	
<60	21(58.3)	41(69.5)	42(66.7)	
Differentiation				0.711[a]
Well	4(11.1)	3(5.1)	3(4.8)	
Moderate	8(22.2)	17(28.8)	19(30.1)	
Poor or undifferentiation	24(66.7)	39(66.1)	41(65.1)	
Location				0.537[a]
Cardia	5(13.9)	13(22.0)	20(31.8)	
Body	6(16.6)	11(18.7)	11(17.5)	
Antrum	23(63.9)	32(54.2)	28(44.4)	
Whole	2(5.6)	3(5.1)	4(6.3)	
Lauren type				0.28[b]
Intestinal type	19(52.8)	31(52.5)	25(39.7)	
Diffuse or mixed type	17(47.2)	28(47.5)	38(60.3)	
CEA				0.097[a]
Elevated	1(2.8)	10(16.9)	9(14.3)	
Normal	35(97.2)	49(83.1)	54(85.7)	
CA19-9				0.542[a]
Elevated	2(5.6)	5(8.5)	8(12.7)	
Normal	34(94.4)	54(91.5)	55(87.3)	
Depth of invasion				0.006[a]
T1	12(33.3)	11(18.6)	4(6.3)	
T2	4(11.1)	7(11.9)	3(4.8)	
T3	6(16.7)	6(10.2)	7(11.1)	
T4a	12(33.3)	25(42.4)	31(49.2)	
T4b	2(5.6)	10(16.9)	18(28.6)	
Lymph node metastasis				0.001[a]
N0	21(58.4)	24(40.7)	13(20.6)	
N1	8(22.2)	9(15.3)	9(14.3)	
N2	4(11.1)	12(20.2)	11(17.5)	
N3a	3(8.3)	7(11.9)	19(30.2)	
N3b	0(0)	7(11.9)	11(17.4)	
Distant metastasis				0.150[a]
Yes	0(0)	1(1.7)	5(7.9)	
No	36(100)	58(98.3)	58(92.1)	
TNM stage				<0.001[a]
I	13(36.1)	17(28.8)	4(6.3)	

II	12(33.3)	9(15.3)	9(14.3)
III	11(30.6)	32(54.2)	45(71.4)
IV	0(0)	1(1.7)	5(8.0)

Variables are in n (%), [a]: *P* values are two-tailed from Fisher's exact test, [b]: *P* values are two-tailed from $\chi 2$ test.

Supplementary Table 7. Clinical characteristics of patients according to the RS in the internal validation cohort 2.

Variables	Internal validation cohort 2 (n = 522)			
	RS-L(%)	RS-M(%)	RS-H(%)	*P*
Gender				0.076[b]
Male	95(64.6)	119(66.5)	147(75.0)	
Female	52(35.4)	60(33.5)	49(25.0)	
Size				0.19[b]
≥4cm	41(27.9)	59(33.0)	73(37.2)	
<4cm	106(72.1)	120(67.0)	123(62.8)	
Age				0.36[b]
≥60	52(35.4)	77(43.0)	80(40.8)	
<60	95(64.6)	102(57.0)	116(59.2)	
Differentiation				0.99[b]
Well	27(18.4)	29(16.2)	33(16.8)	
Moderate	33(22.4)	40(22.3)	44(22.4)	
Poor or undifferentiation	87(59.2)	110(61.5)	119(60.8)	
Location				0.004[b]
Cardia	17(11.6)	30(16.8)	20(10.2)	
Body	29(19.7)	29(16.2)	37(18.9)	
Antrum	95(64.6)	111(62.0)	111(56.6)	
Whole	6(4.1)	9(5.0)	28(14.3)	
Lauren type				0.275[b]
Intestinal type	72(49.0)	84(46.9)	80(40.8)	
Diffuse or mixed type	75(51.0)	95(53.1)	116(59.2)	
CEA				0.03[b]
Elevated	9(6.1)	17(9.5)	29(14.8)	
Normal	138(93.9)	162(90.5)	167(85.2)	
CA19-9				0.129[b]
Elevated	17(11.6)	34(19.0)	37(18.9)	
Normal	130(88.4)	145(81.0)	159(81.1)	
Depth of invasion				<0.001[a]
T1	52(35.4)	45(25.1)	47(24.0)	
T2	32(21.8)	35(19.5)	14(7.1)	
T3	6(4.0)	6(3.4)	3(1.5)	
T4a	42(28.6)	63(35.2)	60(30.6)	
T4b	15(10.2)	30(16.8)	72(36.8)	
Lymph node metastasis				0.02[b]
N0	82(55.8)	75(41.9)	88(44.9)	
N1	34(23.1)	49(27.4)	35(17.9)	
N2	14(9.5)	23(12.8)	25(12.8)	
N3a	10(6.8)	26(14.5)	34(17.3)	
N3b	7(4.8)	6(3.4)	14(7.1)	
Distant metastasis				0.005[b]
Yes	9(6.1)	10(5.6)	28(14.3)	
No	138(93.9)	169(94.4)	168(85.7)	
TNM stage				<0.001[b]

I	53(36.1)	39(21.8)	37(18.8)
II	38(25.9)	50(27.9)	36(18.4)
III	47(32.0)	80(44.7)	95(48.5)
IV	9(6.0)	10(5.6)	28(14.3)

Variables are in n (%). [a]: *P* values are two-tailed from Fisher's exact test, [b]: *P* values are two-tailed from $\chi 2$ test.

Supplementary Table 8. Clinical characteristics of patients according to the RS in the external validation cohort.

Variables	External validation cohort (n = 1231)			
	RS-L(%)	RS-M(%)	RS-H(%)	*P*
Gender				<0.001[b]
Male	234(63.6)	255(65.2)	357(75.6)	
Female	134(36.4)	136(34.8)	115(24.4)	
Size				<0.001[b]
≥4cm	154(41.8)	240(61.4)	357(75.6)	
<4cm	214(58.2)	151(38.6)	115(24.4)	
Age				0.212[b]
≥60	150(40.8)	162(41.4)	218(46.2)	
<60	218(59.2)	229(58.6)	254(53.8)	
Differentiation				0.004[a]
Well	13(3.5)	1(0.3)	6(1.3)	
Moderate	48(13.0)	66(16.9)	81(17.1)	
Poor or undifferentiation	307(83.5)	324(82.8)	385(81.6)	
Location				<0.001[b]
Cardia	93(25.3)	129(33.0)	197(41.7)	
Body	72(19.6)	87(22.3)	90(19.1)	
Antrum	199(54.1)	158(40.4)	146(30.9)	
Whole	4(1.0)	17(4.3)	39(8.3)	
Lauren type				0.849[b]
Intestinal type	125(34.0)	138(35.3)	158(33.5)	
Diffuse or mixed type	243(66.0)	253(64.7)	314(66.5)	
CEA				<0.001[b]
Elevated	49(13.3)	77(19.7)	118(25.0)	
Normal	319(86.7)	314(80.3)	354(75.0)	
CA19-9				<0.001[b]
Elevated	41(11.1)	64(16.4)	137(29.0)	
Normal	327(88.9)	327(83.6)	335(71.0)	
Depth of invasion				<0.001[b]
T1	89(24.2)	41(10.5)	26(5.5)	
T2	60(16.3)	56(14.3)	21(4.5)	
T3	83(22.6)	102(26.1)	86(18.2)	
T4a	120(32.6)	168(43.0)	280(59.3)	
T4b	16(4.3)	24(6.1)	59(12.5)	
Lymph node metastasis				<0.001[b]
N0	166(45.1)	137(35.0)	103(21.8)	
N1	59(16.0)	70(17.9)	70(14.8)	
N2	58(15.8)	57(14.6)	98(20.8)	
N3a	56(15.2)	89(22.8)	120(25.4)	
N3b	29(7.9)	38(9.7)	81(17.2)	
Distant metastasis				<0.001[b]
Yes	24(6.5)	33(8.4)	84(17.8)	
No	344(93.5)	358(91.6)	388(82.2)	
TNM stage				<0.001[b]
I	113(30.7)	65(16.6)	38(8.1)	

II	114(31.0)	117(29.9)	79(16.7)
III	117(31.8)	176(45.1)	271(57.4)
IV	24(6.5)	33(8.4)	84(17.8)

Variables are in n (%). [a]: *P* values are two-tailed from Fisher's exact test, [b]: *P* values are two-tailed from $\chi 2$ test.

Supplementary Table 9. Clinical characteristics of patients according to the RS in the immunotherapy cohorts.

Variables	SMU and GPHCM cohorts (n = 121)			
	RS-L(%)	RS-M(%)	RS-H(%)	*P*
Gender				0.525[b]
Male	16(53.3)	18(45.0)	29(56.9)	
Female	14(46.7)	22(55.0)	22(43.1)	
Age				0.799[b]
≥60	11(36.7)	15(37.5)	22(43.1)	
<60	19(63.3)	25(62.5)	29(56.9)	
TNM stage				0.625[a]
II	1(3.3)	2(5.0)	2(3.9)	
III	9(30.0)	16(40.0)	13(25.5)	
IV	20(66.7)	22(55.0)	36(70.6)	
Treatment line (Immunotherapy)				0.949[b]
First Line	8(26.7)	13(32.5)	13(25.5)	
Second line	12(40.0)	16(40.0)	21(41.2)	
Third line	10(33.3)	11(27.5)	17(33.3)	
Treatment response				<0.001[b]
OR	17(56.7)	16(40.0)	5(9.8)	
SD	4(13.3)	11(27.5)	11(21.6)	
PD	9(30.0)	13(32.5)	35(68.6)	

OR: objective response, SD: stable disease, PD: progressive disease, variables are in n (%), [a]: *P* values are two-tailed from Fisher's exact test, [b]: *P* values are two-tailed from $\chi 2$ test.

Supplementary Table 10. AUCs of the RS and the selected features in predicting NLR status in the training cohort, internal validation cohort 1, and external validation cohort 1.

Variables	Training cohort		Internal validation cohort 1		External validation cohort 1	
	AUC (95%CI)	*P*	AUC (95%CI)	*P*	AUC (95%CI)	*P*
RS	0.861 (0.807-0.915)	<0.001	0.799 (0.721-0.878)	<0.001	0.807 (0.702-0.912)	<0.001
peri-GLSZM_ZSV	0.471 (0.381-0.560)	0.521	0.423 (0.318-0.529)	0.157	0.495 (0.349-0.641)	0.944
peri-GLRLM_RLV	0.523 (0.433-0.614)	0.61	0.479 (0.373-0.585)	0.697	0.560 (0.408-0.712)	0.42
peri-NGTDM_Busyness	0.717 (0.639-0.796)	<0.001	0.727 (0.636-0.819)	<0.001	0.643 (0.495-0.791)	0.054
intro-NGTDM_Busyness_1.0	0.734 (0.657-0.811)	<0.001	0.730 (0.639-0.822)	<0.001	0.684 (0.548-0.820)	0.013
intro-NGTDM_Strength_1.0	0.199 (0.133-0.265)	<0.001	0.216 (0.134-0.298)	<0.001	0.253 (0.131-0.375)	0.001
peri-GLRLM_LRHGE_2.5	0.626 (0.540-0.712)	0.006	0.549 (0.444-0.655)	0.362	0.638 (0.500-0.775)	0.064
intro-GLCM_ClusterProminence_1.0	0.292 (0.213-0.372)	<0.001	0.314 (0.216-0.411)	0.001	0.311 (0.178-0.444)	0.011
peri-GLCM_Correlation_1.0	0.707 (0.626-0.789)	<0.001	0.616 (0.511-0.720)	0.033	0.622 (0.481-0.763)	0.101
intro-GLRLM_GLN	0.649 (0.564-0.733)	0.001	0.638 (0.536-0.740)	0.011	0.667 (0.530-0.804)	0.025
peri-perimeter	0.787 (0.717-0.857)	<0.001	0.774 (0.691-0.858)	<0.001	0.720 (0.594-0.845)	0.003

RS: Radiomics score, *P* values are two-sided from Delong's test.

Supplementary Table 11. Univariate association of RS, clinicopathological characteristics with disease-free and overall survival in the training cohort.

Variables	Disease-free survival		Overall survival	
	HR (95%CI)	*P*	HR (95%CI)	*P*
RS	3.394 (2.496-4.615)	<0.0001	3.034 (2.200-4.182)	<0.0001
Age (years) (≥60 vs. <60)	1.018 (0.691-1.500)	0.928	0.974 (0.627-1.512)	0.906
Gender (Male vs. female)	1.085 (0.731-1.611)	0.686	1.374 (0.867-2.178)	0.176
Tumor size (>4 cm vs. ≤4 cm)	1.840 (1.247-2.717)	0.002	1.872 (1.202-2.916)	0.006
Tumor location	1.041 (0.834-1.300)	0.722	1.044 (0.813-1.341)	0.734
Differentiation	1.476 (1.078-2.022)	0.015	1.571 (1.091-2.262)	0.015
Lauren type	1.287 (0.874-1.895)	0.202	1.313(0.846-2.036)	0.224
CEA (Elevated vs. normal)	1.999 (1.188-3.362)	0.009	2.590 (1.498-4.476)	0.001
CA19-9 (Elevated vs. normal)	1.550 (0.922-2.606)	0.098	1.264 (0.685-2.331)	0.454
Depth of invasion	1.723 (1.478-2.009)	<0.0001	1.757 (1.470-2.100)	<0.0001
Lymph node metastasis	1.585 (1.401-1.793)	<0.0001	1.645 (1.430-1.893)	<0.0001
Distant metastasis	6.626 (4.065-10.800)	<0.0001	4.725 (1.882-11.864)	<0.0001

RS: Radiomics score, HR: hazard ratio, *P* values reported are two-tailed from Cox proportional hazard regression analyses.

Supplementary Table 12. Univariate association of RS, clinicopathological characteristics with disease-free and overall survival in the internal validation cohort 1.

Variables	Disease-free survival		Overall survival	
	HR (95%CI)	*P*	HR (95%CI)	*P*
RS	3.235 (2.195-4.768)	<0.0001	3.027 (2.033-4.508)	<0.0001
Age (years) (≥60 vs. <60)	0.969 (0.616-1.523)	0.891	1.123 (0.700-1.801)	0.631
Gender (Male vs. female)	1.254 (0.772-2.039)	0.361	1.228 (0.729-2.067)	0.44
Tumor size (>4 cm vs. ≤4 cm)	2.464 (1.557-3.899)	<0.0001	2.242 (1.385-3.631)	0.001
Tumor location	0.836 (0.665-1.052)	0.127	0.832 (0.651-1.064)	0.142
Differentiation	1.127 (0.778-1.631)	0.527	1.040 (0.710-1.522)	0.841
Lauren type	1.063 (0.694-1.627)	0.78	0.988 (0.628-1.554)	0.957
CEA (Elevated vs. normal)	1.729 (0.973-3.073)	0.062	1.695 (0.913-3.146)	0.095
CA19-9(Elevated vs. normal)	1.639 (0.867-3.100)	0.128	1.876 (0.986-3.571)	0.055
Depth of invasion	1.654 (1.392-1.966)	<0.0001	1.652 (1.377-1.981)	<0.0001
Lymph node metastasis	1.850 (1.568-2.182)	<0.0001	1.741 (1.465-2.068)	<0.0001
Distant metastasis	6.961 (2.876-16.847)	<0.0001	3.256 91.290-8.215)	0.012

RS: Radiomics score, HR: hazard ratio, *P* values reported are two-tailed from Cox proportional hazard regression analyses.

Supplementary Table 13. Univariate association of RS, clinicopathological characteristics with disease-free and overall survival in the internal validation cohort 2.

Variables	Disease-free survival		Overall survival	
	HR (95%CI)	*p*	HR (95%CI)	*p*
RS	1.694 (1.440-1.993)	<0.0001	1.767 (1.479-2.110)	<0.0001
Age (years) (≥60 vs. <60)	1.091 (0.862-1.382)	0.47	1.301 (1.002-1.689)	0.048
Gender (Male vs. female)	0.885 (0.692-1.131)	0.328	0.903 (0.686-1.190)	0.469
Tumor size (>4 cm vs. ≤4 cm)	1.182 (0.928-1.504)	0.176	1.243 (0.951-1.625)	0.111
Tumor location	1.080 (0.925-1.262)	0.328	1.081 (0.911-1.283)	0.374
Differentiation	1.189 (1.017-1.390)	0.03	1.322 (1.101-1.588)	0.003
Lauren type	1.202 (0.951-1.520)	0.124	1.102 (0.848-1.430)	0.468
CEA (Elevated vs. normal)	2.512 (1.822-3.463)	<0.0001	2.648 (1.855-3.778)	<0.0001
CA19-9 (Elevated vs. normal)	2.635 (2.005-3.463)	<0.0001	2.530 (1.864-3.435)	<0.0001
Depth of invasion	1.514 (1.411-1.624)	<0.0001	1.538 (1.420-1.667)	<0.0001
Lymph node metastasis	1.357 (1.241-1.484)	<0.0001	1.383 (1.253-1.526)	<0.0001
Distant metastasis	4.876 (3.506-6.780)	<0.0001	4.148 (2.910-5.911)	<0.0001

RS: Radiomics score, HR: hazard ratio, *P* values reported are two-tailed from Cox proportional hazard regression analyses.

Supplementary Table 14. Univariate association of RS, clinicopathological characteristics with disease-free and overall survival in the external validation cohort.

Variables	Disease-free survival		Overall survival	
	HR (95%CI)	*p*	HR (95%CI)	*p*
RS	1.860 (1.626-2.128)	<0.0001	1.883 (1.646-2.153)	<0.0001
Age (years) (≥60 vs. <60)	1.366 (1.124-1.660)	0.002	1.347 (1.108-1.639)	0.003
Gender (Male vs. female)	1.094 (0.884-1.355)	0.391	1.079 (0.871-1.336)	0.485
Tumor size (>4 cm vs. ≤4 cm)	2.020 (1.624-2.514)	<0.0001	1.080 (1.668-2.594)	<0.0001
Tumor location	0.883 (0.795-0.981)	0.02	0.889 (0.800-0.987)	0.027
Differentiation	1.275 (1.001-1.624)	0.049	1.297 (1.016-1.655)	0.037
Lauren type	1.480 (1.191-1.841)	0.001	1.512 (1.214-1.883)	<0.001
CEA (Elevated vs. normal)	1.762 (1.417-2.190)	<0.001	1.771 (1.424-2.203)	<0.001
CA199 (Elevated vs. normal)	2.440 (1.980-3.006)	<0.0001	2.440 (1.980-3.008)	<0.0001
Depth of invasion	1.613 (1.489-1.747)	<0.0001	1.625 (1.499-1.763)	<0.0001
Lymph node metastasis	1.661 (1.545-1.786)	<0.0001	1.676 (1.558-1.802)	<0.0001
Distant metastasis	4.073 (3.219-5.154)	<0.0001	4.265 (3.367-5.403)	<0.0001

RS: Radiomics score, HR: hazard ratio, *P* values reported are two-tailed from Cox proportional hazard regression analyses.

Supplementary Table 15. Comparing the prediction power of the integrated nomogram with RS and TNM stage in the training and validation cohorts.

Variable	Disease-free survival	Overall survival
	C-Index (95% CI)	C-Index (95% CI)
Training cohort		
Nomogram	0.784 (0.745-0.823)	0.791 (0.749-0.834)
RS	0.716 (0.675-0.757)	0.736 (0.689-0.783)
TNM Stage	0.726 (0.687-0.765)	0.721 (0.678-0.764)
Internal Validation cohort 1		
Nomogram	0.779 (0.730-0.828)	0.766 (0.713-0.819)
RS	0.676 (0.623-0.729)	0.670 (0.613-0.727)
TNM Stage	0.715 (0.690-0.740)	0.717 (0.689-0.744)
Internal Validation cohort 2		
Nomogram	0.756 (0.731-0.781)	0.750 (0.720-0.780)
RS	0.620 (0.587-0.653)	0.630 (0.595-0.665)
TNM Stage	0.715 (0.690-0.740)	0.718 (0.689-0.747)
External Validation cohort		
Nomogram	0.762 (0.742-0.782)	0.748 (0.726-0.770)
RS	0.636 (0.609-0.663)	0.638 (0.611-0.665)
TNM Stage	0.729 (0.709-0.749)	0.732 (0.712-0.752)

RS: Radiomics score.

Supplementary Table 16. Antibody sources and staining conditions.

Markers	Main target	Antibody source	Species	Dilution	DAB dyeing time	Antigen Retrieval	Cellular localization
CD8	Cytotoxic T lymphocyte	NeoMarker, clone SP16	Rabbit monoclonal	1:200	1.5 min	Citrate buffer (pH 6.0) microwave 20min	Membranous
CD66b	Neutrophil	BD Pharmingen	Mouse monoclonal	1:200	1.0 min	Citrate buffer (pH 6.0) microwave 20min	Membranous

min: minute; sec: second. DAB: diaminobenzidine.

Supplementary Table 17. Imaging Biomarker Standardization Initiative (IBSI) reporting structure of the study.

Patients	
Region of interest	CT positive lesion in stomach
Patient Preparation	Patients were required to drink enough water before CT examination to ensure sufficient distention of gastric cavity in CT images
Computed tomography (CT) developing agent	iodinated contrast material
Acquisition and Reconstruction	
Protocol	The acquisition parameters are as follows: 120 kV; 150-190 mAs; 0.5- or 0.4-second rotation time; field of view, 350×350 mm; matrix, 512×512. After routine non-enhanced CT, arterial and portal venous-phase contrast-enhanced CT were performed after delays of 28 s and 60 s following intravenous administration of 90 - 100 ml of iodinated contrast material (Ultravist 370, Bayer Schering Pharma, Berlin, Germany) at a rate of 3.0 or 3.5 ml/s with a pump injector (Ulrich CT Plus 150, Ulrich Medical, Ulm, Germany). Portal venous phase CT images (thickness: range from 1.25 mm to 7.5 mm) were retrieved from the picture archiving and communication system (PACS) (Carestream, Canada) for image feature extraction because of well differentiation of the tumor tissue from the adjacent tissue.
Scanner type	multidetector row CT systems
Delineation	
Software	ITK-SNAP software (version 3.8; www.itksnap.org).
ROI definition	Standard 2D ROI tools
Number of experts	2 + 1 (2 experienced radiologists participated in independent delineations, followed by 1 senior radiologist cross-validation if necessary)
Reference image	CT
Radiomics feature extraction	
Software	Matlab R2016a (The MathWorks Inc.)
Package	radiomics analysis package (https://github.com/yumingjiang/GC_RADIOMICS-.git)
Method	Reads the DICOM content of a single directory; Equal-probability quantization on the region of interest (ROI); computes Lloyd-Max quantization on the region of interest (ROI) of an input volume; computes uniform quantization on the region of interest (ROI) of an input volume; applies the intensity normalization scheme;

	Computation of the smallest box containing region of interest (ROI), if necessary (ROIbox); Wavelet band-pass filtering (WBPF); Isotropic resampling; Quantization of intensity dynamic range.
Discretization	Bin width and LoG filters
Bin width	25 for CT
Kernels of the filter	Gaussian spatial band-pass filter (∇2G)
Biomarker set	intensity features, shape features, gray Level Co-occurrence Matrix-based (GLCM) features, gray Level Run Length Matrix-based (GLRLM) features, gray Level Size Zone Matrix-based (GLSZM) features and neighborhood Gray Tone Difference Matrix–based (NGTDM) features.
Exclusion criteria	ICC smaller than 0.75

Supplementary Reference

1. Jiang Y, Zhang Q, Hu Y, et al. ImmunoScore Signature: A Prognostic and Predictive Tool in Gastric Cancer. *Ann Surg*. 2018;267:504-513.
2. Jiang Y, Xie J, Han Z, et al. Immunomarker Support Vector Machine Classifier for Prediction of Gastric Cancer Survival and Adjuvant Chemotherapeutic Benefit. *Clin Cancer Res*. 2018;24:5574-5584.
3. Jiang Y, Liu W, Li T, et al. Prognostic and Predictive Value of p21-activated Kinase 6 Associated Support Vector Machine Classifier in Gastric Cancer Treated by 5-fluorouracil/Oxaliplatin Chemotherapy. *EBioMedicine*. 2017;22:78-88.
